# The crystal structures of {*Ln*Cu_5_}^3+^ (*Ln* = Gd, Dy and Ho) 15-metallacrown-5 complexes and a reevaluation of the isotypic Eu^III^ analogue

**DOI:** 10.1107/S205698901900999X

**Published:** 2019-07-19

**Authors:** Anna Pavlishchuk, Dina Naumova, Matthias Zeller, Sebastian Calderon Cazorla, Anthony W. Addison

**Affiliations:** a Department of Chemistry, Taras Shevchenko National University of Kyiv, Volodymyrska str. 62, Kyiv, 01601, Ukraine; b L.V. Pisarzhevskii Institute of Physical Chemistry of the National Academy of Sciences of the Ukraine, Prospect Nauki 31, Kyiv 03028, Ukraine; c Department of Chemistry, Purdue University, 560 Oval Drive, West Lafayette, IN 47907-2084, USA; d Department of Chemistry, Drexel University, Philadelphia, PA 19104-2816, USA

**Keywords:** crystal structure, lanthanide(III), hydroxamates, heteropolynuclear complex, metallacrown

## Abstract

Three new isotypic heteropolynuclear complexes, namely penta­aqua­carbonato­penta­kis­(glycinehydroxamato)nitrato­penta­copper(II)lanthanide(III) *x*-hydrate, [*Ln*Cu_5_(GlyHA)_5_(CO_3_)(NO_3_)(H_2_O)_5_]·*x*H_2_O (GlyHA^2−^ is glycine­hydrox­amate, *N*-hy­droxy­glycinamidate or amino­aceto­hydroxamate, C_2_H_4_N_2_O_2_^2−^), with lanthanide(III) (*Ln* ^III^) = gadolinium (Gd, **1**, *x* = 3.5), dysprosium (Dy, **2**, *x* = 3.28) and holmium (Ho, **3**, *x* = 3.445), within a 15-metallacrown-5 class were obtained on reaction of lanthanide(III) nitrate, copper(II) acetate and sodium glycinehydroxamate. Complexes **1**–**3** contain five copper(II) ions and five bridging GlyHA^2−^ anions, forming a [CuGlyHA]_5_ metallamacrocyclic core. The *Ln* ^III^ ions are coordinated to the metallamacrocycle through five O-donor hydroxamates. The electroneutrality of complexes **1**–**3** is achieved by a bidentate carbonate anion coordinated to the *Ln* ^III^ ion and a monodentate nitrate anion coordinated apically to one of the copper(II) ions of the metallamacrocycle. The lattice parameters of complexes **1**–**3** are similar to those previously reported for an Eu^III^–Cu^II^ 15-metallacrown-5 complex with glycine­hydroxamate of proposed composition [EuCu_5_(GlyHA)_5_(OH)(NO_3_)_2_(H_2_O)_4_]·3.5H_2_O [Stemmler *et al.* (1999 ▸). *Inorg. Chem.* **38**, 2807–2817]. High-quality X-ray data obtained for **1**–**3** have allowed a re-evaluation of the X-ray data solution proposed earlier for the EuCu_5_ complex and suggest that the formula is actually [EuCu_5_(GlyHA)_5_(CO_3_)(NO_3_)(H_2_O)_5_]·3.5H_2_O.

## Abstract 2

The crystal structures of three new isomorphous 3*d*–4*f* 15-metallacrown-5 complexes, [*Ln*Cu_5_(GlyHA)_5_(CO_3_)(NO_3_)(H_2_O)_5_]·*x*H_2_O (*Ln* ^III^ = Gd (**1**, *x* = 3.5); Dy (**2**, *x* = 3.28) and Ho (**3**, *x* = 3.45)), were determined. Structural details of the previously reported isotypic Eu^III^ analogue were re­inter­preted.

## Chemical context   

The numerous studies of 3*d*–4*f* metallamacrocyclic complexes in the last few decades arise from their potentially inter­esting catalytic (Griffiths & Kostakis, 2018 ▸), luminescence (Jankolovits *et al.*, 2011 ▸; Li *et al.*, 2017 ▸) and magnetic properties (Dhers *et al.*, 2016 ▸; Zangana *et al.*, 2014 ▸). In addition, a number of heteropolynuclear metallacrown complexes have been shown to possess single mol­ecule magnetic (SMM) behaviour (Ostrowska *et al.*, 2016 ▸; Wang *et al.*, 2019 ▸), bright luminescence with high quantum yields (Nguyen *et al.*, 2018 ▸; Martinić *et al.*, 2017 ▸) and the ability to serve as building blocks for the generation of supra­molecular assemblies and coordination polymers (Pavlishchuk *et al.*, 2014 ▸, 2017*b* ▸). The utilization of heteronuclear cationic 15-metallacrown-5 complexes as initial building blocks has also led to porous structures that are able to absorb various guest mol­ecules (Lim *et al.*, 2010 ▸). The selection of the initial building blocks with labile counter-anions (*e.g.* nitrates) is crucial for the creation of coordination polymers with porous structures. Some of the products of the reactions between glycine­hydroxamate-derived 15-metallacrown-5 complexes and polycarboxyl­ates contain carbonate anions (Pavlishchuk *et al.*, 2014 ▸, 2017*b* ▸), which block the apical positions of the *Ln* ^III^ ions and lead to the formation of discrete assemblies instead of coordination polymers. The presence of carbonate anions in such complexes was associated with the capture of atmospheric carbon dioxide (Pavlishchuk *et al.*, 2014 ▸, 2017*b* ▸).
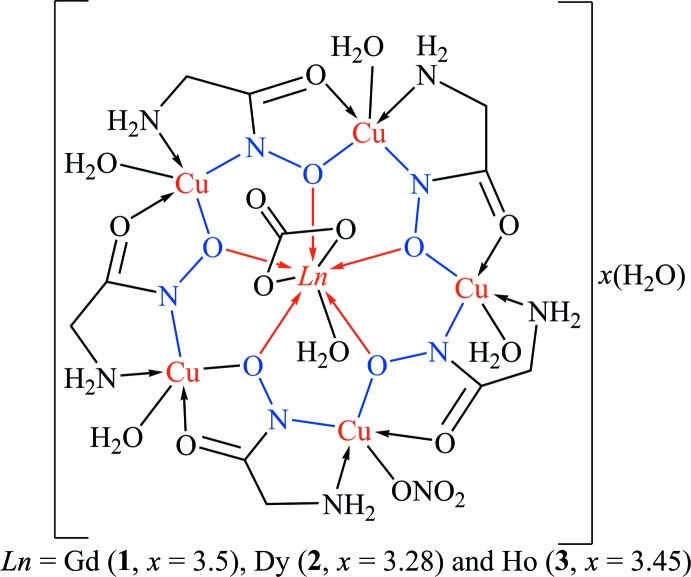


The first examples of 15-metallacrown-5 complexes were reported by Pecoraro and coworkers in 1999 (Stemmler *et al.*, 1999 ▸). One of the reported complexes, a Eu^III^–Cu^II^ glycine­hydroxamate metallacrown, contains the [CuGlyHA]_5_ core and encapsulates an Eu^III^ ion through coordination to five hydroxamate oxygen atoms. It was reported that the positive charge of the 15-metallacrown-5 unit, [EuCu_5_(GlyHA)_5_]^3+^, was partly compensated by two nitrate anions coordinated to copper(II) and europium(III). To fully compensate the positive charge of the [EuCu_5_(GlyHA)_5_]^3+^ unit, an oxygen species coordinated apically to Eu^III^ was assigned as a hydroxide anion, to give a reported overall composition [EuCu_5_(GlyHA)_5_(OH)(NO_3_)_2_(H_2_O)_4_]·3.5H_2_O. The high *R*-factor of 12.3% for the structure was attributed to the presence of disordered water mol­ecules in the crystal structure (Stemmler *et al.*, 1999 ▸). We report here the crystal structures of three isostructural 15-metallacrown-5 complexes, [*Ln*Cu_5_(GlyHA)_5_(CO_3_)(NO_3_)(H_2_O)_5_]·*x*H_2_O (*Ln* ^III^ = Gd (**1**, *x* = 3.5); Dy (**2**, *x* = 3.28) and Ho (**3**, *x* = 3.45)). Based on the high-quality diffraction data collected for **1**–**3**, a new formula of [EuCu_5_(GlyHA)_5_(CO_3_)(NO_3_)(H_2_O)_5_]·3.5H_2_O is proposed for the previously reported Eu compound.

## Structural commentary and supra­molecular features   

**Figure 1:**
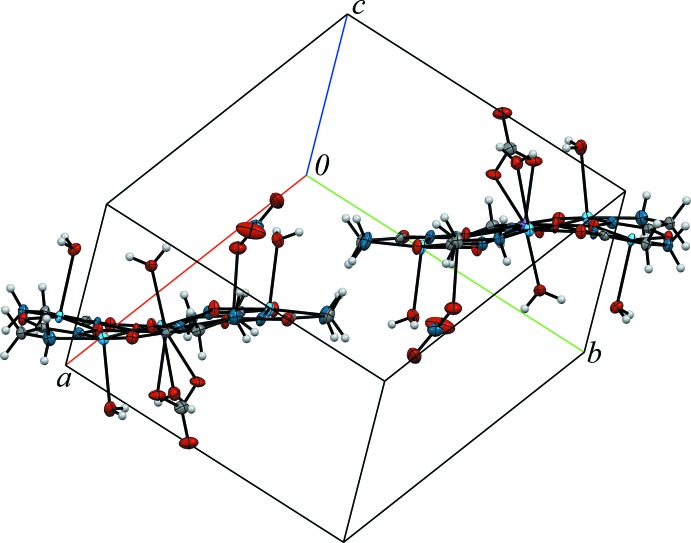
The metallacrown content of a unit cell of complex **1**, showing the relationship of two mol­ecular units through an inversion center. Non-coordinated water mol­ecules and disorder of the carbonate moiety have been omitted for clarity.

Complexes **1**–**3**, and the previously reported europium analogue (Stemmler *et al.*, 1999 ▸), are isotypic based on the unit-cell parameters obtained (Table 1 ▸) and the structure refinement results. The *b* and *c* lattice parameters and the unit-cell volumes for these complexes decrease slightly across the lanthanide series as a result of the lanthanide contraction (Pavlishchuk *et al.*, 2011 ▸, 2017*b* ▸, 2018 ▸; Stemmler *et al.*, 1999 ▸; Zaleski, *et al.*, 2011 ▸). All four compounds crystallize in the space group *P* 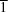 and contain two mol­ecular metallamacrocyclic complexes per unit cell related to each other through a centre of inversion (Fig. 1 ▸). For the convenience of structure description, a common atom-numbering scheme is adopted for **1**–**3**.

**Figure 2:**
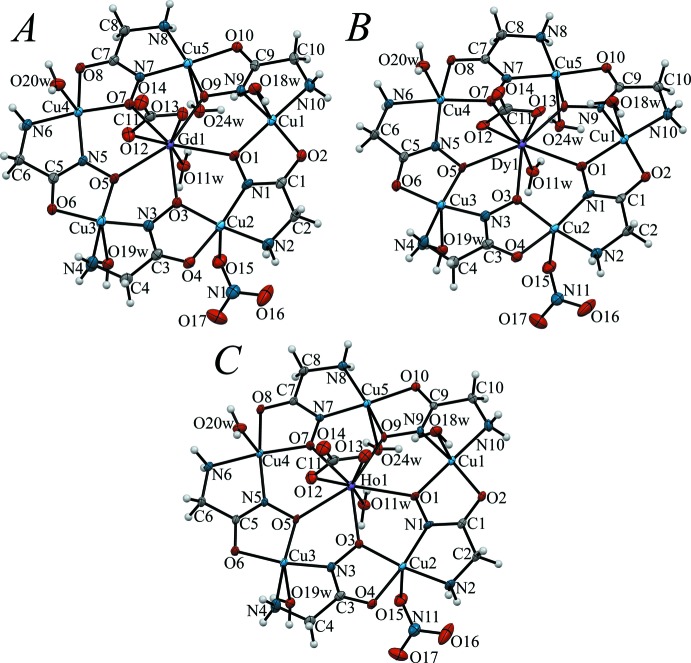
Structures of the metallamacrocyclic cores [*Ln*Cu_5_(GlyHA)_5_(CO_3_)(NO_3_)(H_2_O)_5_] in **1** (Gd, *A*), **2** (Dy, *B*) and **3** (Ho, *C*). Disorder of the carbonate moiety has been omitted for clarity.

The neutral metallamacrocyclic unit [*Ln*Cu_5_(GlyHA)_5_(CO_3_)(NO_3_)(H_2_O)_5_] in **1**–**3** possesses structural features typical of previously characterized Ln^III^–Cu^II^ hydroxamate 15-metallacrown-5 complexes (Pavlishchuk *et al.*, 2011 ▸, 2017*a* ▸,*b* ▸, 2018 ▸; Stemmler *et al.*, 1999 ▸, Zaleski, *et al.*, 2011 ▸; Katkova *et al.*, 2015*a* ▸,*b* ▸). The metallamacrocyclic core is built from the repeating fragment [CuGlyHA], formed *via* bridging coordin­ation of GlyHA^2−^ dianions to two adjacent copper(II) ions, forming two five-membered chelate rings (Fig. 2 ▸). The coord­ination environment of the copper(II) ions in **1**–**3** consists of two nitro­gen atoms (from amino and hydroxamate groups) and two oxygen atoms (from carbonyl and hydrox­amate groups) in their basal planes. The Cu—O and Cu—N bond lengths in **1**–**3** are typical for hydroxamate metallacrown complexes (Tables 2 ▸–4 ▸ ▸) and are comparable with the values previously reported for the Eu^III^ analogue (Stemmler *et al.*, 1999 ▸). All the copper(II) ions in **1**–**3** are penta­coordinate, with N_2_O_3_ donor sets in slightly distorted square-pyramidal coord­ination arrangements [τ values (Addison *et al.*, 1984 ▸) fall in the range of 0.01–0.20, Tables 5 ▸–7 ▸ ▸]. For all the complexes, a pronounced Jahn-Teller-like distortion is observed, with the Cu—N and Cu—O bonds from glycine­hydroxamate in the plane of the metallacrown unit being substanti­ally shorter than the Cu—O bond of the fifth coordination site, the apical position. This site for the Cu2 atom is in each case occupied by the oxygen atom O15 from the monodentate nitrate anions [Cu2—O15 is 2.469 (3) Å in **1**, 2.464 (3) Å in **2** and 2.463 (3) Å in **3**], while the coordination spheres of the copper(II) ions of Cu1, Cu3, Cu4 and Cu5 ions in **1**–**3** are completed by oxygen atoms of coordinated water mol­ecules. The Cu—Ow bond distances in **1**–**3** range from 2.400 (3) to 2.476 (3) Å. The shorter Cu—O and Cu—N bond lengths within the metallacrown plane of **1**–**3**, on the other hand, range from 1.897 (3) to 2.022 (2) Å.

**Figure 3:**
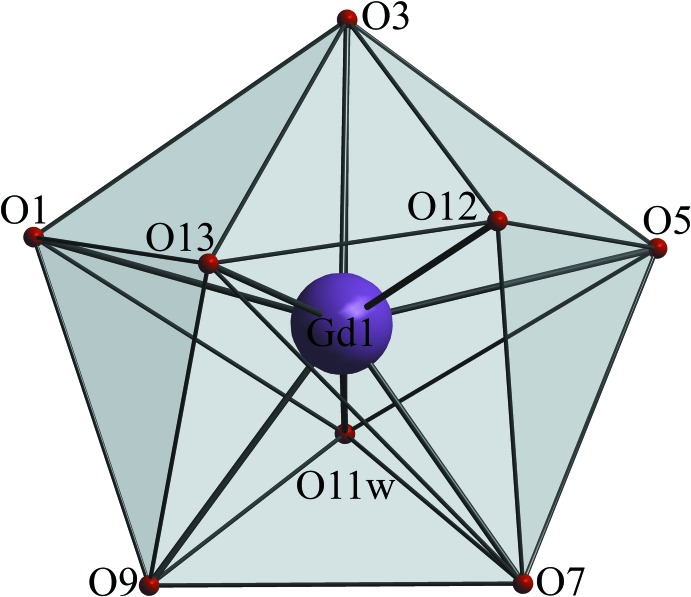
The coordination environment of the Gd^III^ ion in **1**.

The centre of the metallamacrocyclic core [CuGlyHA]_5_ in **1**–**3** is occupied by Gd^III^, Dy^III^ and Ho^III^ ions, respectively, which are coordinated through five hydroxamate oxygen atoms from glycine­hydroxamate. The equatorial *Ln*—O bond distances in **1**–**3** range from 2.381 (2) to 2.484 (2) Å, 2.382 (3) to 2.469 (3) Å and 2.374 (2) to 2.475 (2) Å respectively, paralleling the lanthanide contraction (Table 8 ▸). The Ln^III^ ions in **1**–**3** and the previously reported Eu^III^ analogue are octa­coordinate (Fig. 3 ▸), and the coordination geometry of the Gd^III^, Dy^III^ and Ho^III^ ions in **1**–**3** can be described as triangular dodeca­hedral (*D*_2*d*_), according to *Shape2.1* calculations (Table 9 ▸) (Casanova *et al.*, 2005 ▸).

**Figure 4:**
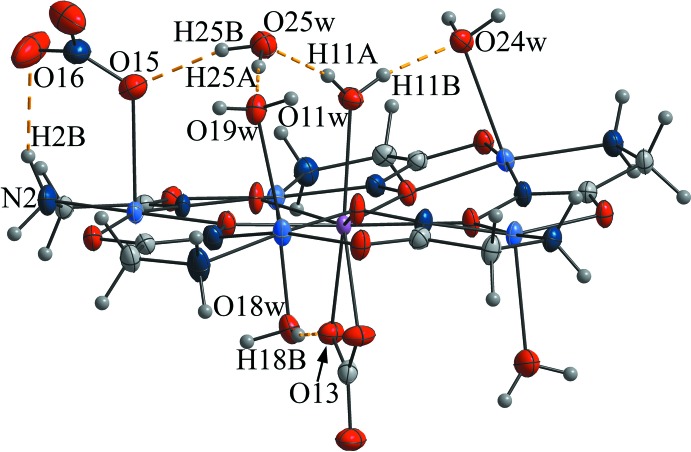
A metallacrown moiety in **1**, showing the involvement of the apically coordinated ligands in hydrogen bonds (shown as dotted lines).

**Figure 5:**
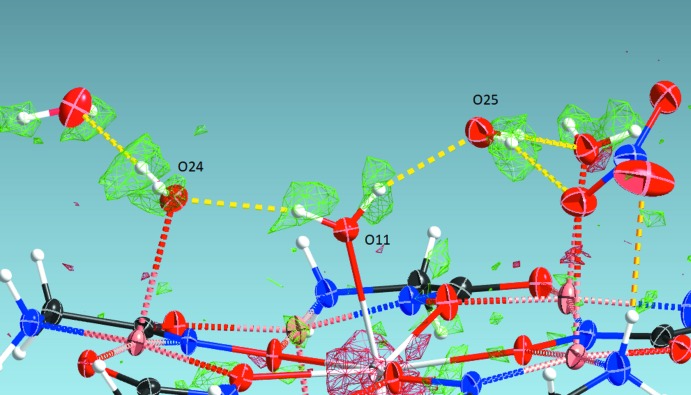
Difference electron density in **2** around the water mol­ecules O11w, O24w and O25w (using the *SHELXLE* GUI for *SHELXL*; Hübschle *et al.*, 2011 ▸). Difference electron densities were calculated with water H atoms removed from the final structural model. H-atom positions, refined as described in the *Refinement* section, are shown. The difference electron-density mesh setting used is 0.28 e Å^−3^.

The two *Ln* ^III^ ion apical positions in **1**–**3** are occupied by oxygen atoms O12 [2.317 (11), 2.27 (2) and 2.304 (16) Å in **1**–**3**] and O13 [2.288 (17), 2.31 (3) and 2.30 (3) Å in **1**–**3**] from the bidentately coordinated carbonate anion, forming the charge-balanced moiety, [*Ln*Cu_5_(GlyHA)_5_(CO_3_)(NO_3_)(H_2_O)_5_]. The apical coordination of carbonate dianions to *Ln* ^III^ ions in 15-metallacrown-5 units has been observed previously and can be associated with the capture of atmos­pheric carbon dioxide (Pavlishchuk *et al.*, 2014 ▸, 2017*b* ▸, 2018 ▸). The inter­pretation of the X-ray data for the previously described isotypic Eu^III^ complex was based on the assumption of bidentate coordin­ation of two nitrates to Eu^III^ ions instead of one nitrate and one carbonate (Stemmler *et al.*, 1999 ▸). The reported *Ln*—O(nitrate) bond distances for the Eu^III^ analogue (Stemmler *et al.*, 1999 ▸) are comparable with values for the *Ln*—O(carbon­ate) bonds observed in **1**–**3**, and the Eu—O donor previously attributed to a hydroxide ion is assigned here as a water mol­ecule. The third apical position of the *Ln* ^III^ ions in **1**–**3**, *trans* to the carbonate anion is occupied by an oxygen donor atom, O11w, from a coordinated water mol­ecule [Gd1—O11w = 2.359 (2), Dy1—O11w = 2.357 (3) and Ho1—O11w = 2.358 (2) Å]. The *Ln*—O bond lengths for terminal *Ln*—OH and *Ln*—OH_2_ are generally very similar, and a clear distinction between water and OH^−^ as the terminal ligand in **1**–**3** and in the previously reported Eu^III^ analogue cannot reliably be made. Moreover, both *Ln*—OH and *Ln*—OH_2_ bond lengths are strongly dependent on the lanthanide ion radius, its coordination number and the coordination geometry (Novitchi *et al.*, 2012 ▸; Yang *et al.*, 2013 ▸; Yi *et al.*, 2013 ▸; Chen *et al.*, 2012 ▸; Wang *et al.*, 2012 ▸; Gao *et al.*, 2011 ▸; Xu *et al.*, 2011 ▸; Dai *et al.*, 2011 ▸). While no definite conclusions can therefore be drawn from the bond distances involving *Ln*—O11, the hydrogen-bonding inter­actions (Tables 10 ▸–12 ▸ ▸) involving O11 are informative. The water mol­ecule, O11w, acts as a hydrogen bonding donor to two water mol­ecules: forming an intra­molecular hydrogen bond with coordinated water mol­ecule O24w, as O11w—H11*B*⋯O24w, and with the non-coordin­ated water mol­ecule, O25w, *via* O11w—H11*A*⋯O25w bond (shown for **1** in Fig. 4 ▸). Hydrogen atoms for all three water mol­ecules are clearly resolved in difference electron-density maps (shown for **2** in Fig. 5 ▸). In **1**–**3**, one of the non-coord­in­ated water mol­ecules, on the O23w site, was refined as partially occupied [occupancy factors 0.499 (11), 0.280 (14) and 0.445 (11), respectively]. Overall, the observed positions of the H atoms as well as the hydrogen-bonding network itself, based on the positions and distances of the involved oxygen atoms, are incompatible with the assumption of the presence of a hydroxide anion, as previously proposed.

The *Ln* ^III^⋯Cu^II^ and Cu^II^⋯Cu^II^ separations in the metallamacrocyclic cores of **1**–**3** and their Eu^III^ analogue (Table 8 ▸) have values typical of hydroxamate 15-metallacrown-5 complexes (Stemmler *et al.*, 1999 ▸). The metallamacrocyclic cores [*Ln*Cu_5_(GlyHA)_5_]^3+^ are almost planar: the average deviations of non-hydrogen atoms from the Cu_5_ mean planes do not exceed 0.2 Å, while the deviations of the *Ln* ions from the Cu_5_ mean planes have values typical for 15-metallacrown-5 complexes. (Pavlishchuk *et al.*, 2011 ▸, 2017*b* ▸, 2018 ▸; Stemmler *et al.*, 1999 ▸; Zaleski, *et al.*, 2011 ▸; Katkova *et al.*, 2015*a* ▸,*b* ▸). The largest deviations from the Cu_5_ mean planes are observed for nitro­gen atoms N8 from amino groups located on the periphery of the metallamacrocyclic [*Ln*Cu_5_(GlyHA)_5_]^3+^ cores (Table 8 ▸).

**Figure 6:**
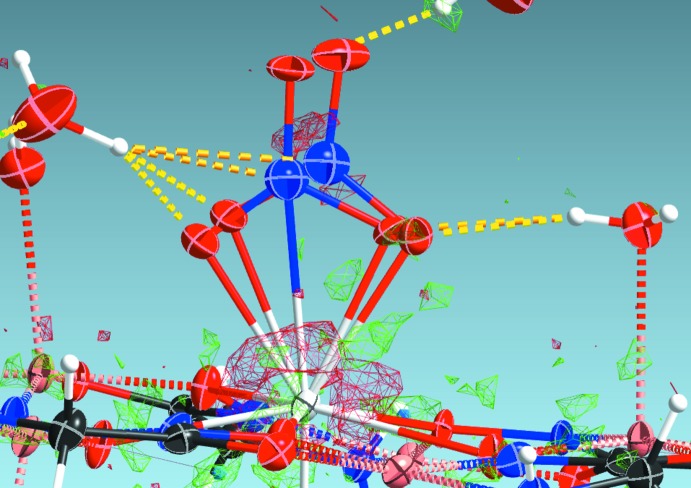
Displacement ellipsoid plot with difference electron density after refinement of the carbonate ion as a ‘nitrate’ in **2** (using the *SHELXLE* GUI for *SHELXL*; Hübschle *et al.*, 2011 ▸). The large thermal parameters for the central atoms, compared to neighbouring oxygen atoms, indicates insufficient available electron density for the presence of a ‘nitro­gen’ atom. The difference electron-density mesh setting used is 0.28 e Å^−3^.

The 15-metallacrown-5 units [*Ln*Cu_5_(GlyHA)_5_]^3+^ in **1**–**3** are non-oligomerised, as is typical for heteropolynuclear 15-metallacrown-5 complexes. The [*Ln*Cu_5_(GlyHA)_5_(CO_3_)(NO_3_)(H_2_O)_5_] fragments are linked to each other through an extended system of hydrogen bonds. Carbonates coordinated to *Ln* ^III^ ions link each [*Ln*Cu_5_(GlyHA)_5_(CO_3_)(NO_3_)(H_2_O)_5_] unit with two adjacent metallamacrocycles through O13^i^⋯H2*A*—N2 [symmetry code: (i) 2 − *x*, 1 − *y*, 1 − *z*] and O13*B*⋯H18*B*—O18w bonds. Nitrate anions in [*Ln*Cu_5_(GlyHA)_5_(CO_3_)(NO_3_)(H_2_O)_5_] also connect each metalla­macro­cyclic unit with neighbouring cations *via* O17^ii^⋯H10*A*—N10 hydrogen bonds [symmetry code: (ii) *x* + 1, *y*, *z*]. In addition, water mol­ecules coordinated to the Cu^II^ ions in **1**–**3** also link {*Ln*Cu_5_}^3+^ cores with adjacent fragments (O18w^*i*ii^⋯H19*B*—O19 [symmetry code: (iii) *x* − 1, *y*, *z*], O18w—H18*A*⋯O4^i^, O19w—H19*A*⋯O8^iv^ [symmetry code: (iv) −*x* + 1, −*y*, −*z*], O20w—H20*A*⋯O10^v^ [symmetry code: (v) −*x* + 2, −y, −z], O20w—H20*B*⋯O14^vi^ [symmetry code: (vi) −*x* + 2, −*y* + 1, −*z*], O24w—H24*A*⋯O2^vii^ [symmetry code: (vii) −*x* + 2, −*y*, −*z* + 1]. Multiple hydrogen bonds connect 15-metallacrown-5 units with non-coordinated water mol­ecules. As mentioned above, one of the the non-coordinated water mol­ecule positions, O23w, is partially occupied, inducing disorder for the nearby carbonate anion, with refined occupancy factors of 0.499 (11), 0.280 (14) and 0.445 (11) in **1**–**3**, respectively (see the *Refinement* section for details). Bond distances within the anion are biased because of the disorder, so no assignment of nitrate *vs* carbonate can be made based on expected N—O or C—O bond distances. However, despite this disorder involving the anions neighbouring the partially occupied water mol­ecule, the nature of the entity as a carbonate anion is clearly resolved in difference electron-density maps. Replacement of the carbonate carbon atom, C11, with a nitro­gen atom results only in a marginal increase in *R* value (*e.g.* 3.10 *vs* 3.07% for compound **2**). Thermal parameters of the ‘nitro­gen’ atoms do however become unreasonably large, compared to the neighbouring oxygen atoms, and a positive residual electron density is clearly visible around the central atoms of the anion when refined as nitro­gen (Fig. 6 ▸).

For the other O_3_ *X* anion, coordinated to Cu4, the opposite observation can be made, and this anion matches the electron density requirements of a nitrate anion.

In summary, we have synthesized three new metallamacrocyclic (*Ln* = Gd, Dy and Ho) complexes with glycine­hydroxamate, which are isotypic with the first representative of the 15-metallacrown-5 family reported in 1999. The better quality of the new structural data allow us to propose an alternative composition [*Ln*Cu_5_(GlyHA)_5_(CO_3_)(NO_3_)(H_2_O)_5_]·*x*H_2_O [*Ln* ^III^ = Gd (**1**, *x* = 3.5), Dy (**2**, *x* = 3.28) and Ho (**3**, *x* = 3.45)] for this series of compounds and the previously reported Eu complex (*x* = 3.5). The cationic charge of the {*Ln*Cu_5_}^3+^ metallamacrocyclic cores in **1**–**3** is compensated by a monodentate nitrate anion coordinated to a Cu^II^ ion and a bidentate carbonate ion linked to the *Ln* ^III^ ions. The presence of capping carbonate anions in metallacrown building blocks can prevent the formation of coordination polymers based on the metallacrown complex.

## Synthesis and crystallization   

Complexes **1**–**3** were prepared and isolated as dark-blue single crystals according to the previously reported procedure for the Eu^III^ analogue, using Gd(NO_3_)_3_·6H_2_O, Dy(NO_3_)_3_·6H_2_O and Ho(NO_3_)_3_·5H_2_O, respectively (Stemmler *et al.*, 1999 ▸).

## Refinement   

Crystal data, data collection and structure refinement details are summarized in Table 13 ▸. The three structures are isotypic and were refined using a common structural model.

All carbon- and nitro­gen-bound H atoms, while observed in difference density maps, were placed in calculated positions, with C—H distances of 0.99 Å and N—H distances of 0.91 Å. All H-atom positions of the ordered water mol­ecules were clearly resolved in difference electron-density maps and their positions were refined with O—H and H⋯H distances restrained to 0.84 (2) and 1.36 (2) Å, respectively. The H-atom positions of the disordered water moieties were further restrained based on hydrogen-bonding considerations. In the final refinement cycles, the partially occupied H atoms were set to ride on their carrier oxygen atoms. *U*_iso_ values of all H atoms were set to a multiple of their respective carrier atom, with *U*_iso_(H) = 1.2*U*_eq_(C/N) or 1.5*U*_eq_(O).

In all three structures, one water mol­ecule position is partially occupied, inducing disorder for the nearby carbonate anion. The two disordered carbonate moieties were restrained to have similar geometries (using *SHELXL* SAME restraints, esd = 0.02 Å). *U^ij^* components of ADPs for disordered atoms closer to each other than 2.0 Å were restrained to be similar within a standard deviation of 0.01 Å^2^ (SIMU restraint of *SHELXL*). In **1** and **3**, the distance of the water oxygen to one of the carbonate oxygen atoms was restrained to be at least 2.80 (2) Å for the moiety that contains the water mol­ecule [2.75 (2) Å for **2**]. Subject to these conditions, the occupancy ratios refined to 0.499 (11) to 0.501 (11) for **1**, 0.280 (14) to 0.720 (14) for **2** and 0.445 (11) to 0.555 (11) for **3**.

There is an indication of additional disorder involving the partially ordered water mol­ecule nearby the carbonate ion. This additional disorder is not well enough resolved to be independently refined and may cause the B alerts in the CIF files of **1**–**3**. We opted to not attempt to refine additional potentially highly ambiguous disorder.

## Supplementary Material

Crystal structure: contains datablock(s) Complex_1, Complex_2, Complex_3, global. DOI: 10.1107/S205698901900999X/cq2031sup1.cif

Structure factors: contains datablock(s) Complex_1. DOI: 10.1107/S205698901900999X/cq2031Complex_1sup2.hkl

Structure factors: contains datablock(s) Complex_2. DOI: 10.1107/S205698901900999X/cq2031Complex_2sup3.hkl

Structure factors: contains datablock(s) Complex_3. DOI: 10.1107/S205698901900999X/cq2031Complex_3sup4.hkl

CCDC references: 1940082, 1940083, 1940084

Additional supporting information:  crystallographic information; 3D view; checkCIF report

## Figures and Tables

**Table 1:** Comparison of single-crystal data and structure refinement details for complexes **1**–**3** with those of their earlier reported Eu^III^ analogue (CCDC127569, Stemmler *et al.*, 1999 ▸)

	CCDC127569	Complex **1**	Complex **2**	Complex **3**
Formula	[EuCu_5_(GlyHA)_5_(CO_3_)(NO_3_)(H_2_O)_5_]·3.5H_2_O	[GdCu_5_(GlyHA)_5_(CO_3_)(NO_3_)(H_2_O)_5_]·3.5H_2_O	[DyCu_5_(GlyHA)_5_(CO_3_)(NO_3_)(H_2_O)_5_]·3.28H_2_O	[HoCu_5_(GlyHA)_5_(CO_3_)(NO_3_)(H_2_O)_5_]·3.445H_2_O
*M* (g mol^−1^)	1186.17	1190.49	1191.77	1197.21
Crystal system	Triclinic	Triclinic	Triclinic	Triclinic
Space group	*P* 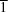	*P* 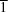	*P* 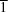	*P* 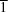
*a* (Å)	11.163 (5)	11.2057 (15)	11.1083 (5)	11.2027 (9)
*b* (Å)	11.524 (4)	11.5054 (15)	11.4991 (5)	11.4955 (9)
*c* (Å)	13.323 (4)	13.2983 (10)	13.2894 (6)	13.2467 (10)
α (°)	93.85 (3)	94.026 (4)	93.9235 (16)	94.001 (3)
β (°)	94.79 (3)	94.942 (3)	94.7713 (17)	94.784 (3)
γ (°)	107.14 (3)	107.558 (3)	107.1470 (17)	107.518 (3)
Volume (Å^3^)	1624.5 (10)	1620.2 (3)	1608.73 (13)	1613.0 (2)
*Z*	2	2	2	2
*T* (K)	293 (2)	150 (2)	150 (2)	150 (2)
Range of data collection	2.53 °<2θ < 26.03°	3.091° < 2θ <33.234°	3.091° <2θ <28.693°	2.544° <2θ <33.243°
ρ_calc_ (g cm^−3^)	2.425	2.440	2.460	2.467
μ (mm^−1^)	5.229	5.353	5.651	5.773
*F*(000)	1168	1170	1170	1174.9
Collected reflections	6344	118155	74286	50443
Reflections unique	6344	12399	8274	12306
*R*_int_	0.1272	0.0560	0.0483	0.0417
Goodness-of-fit on *F* ^2^	1.114	1.050	1.084	1.053
*R*_1_([*I* > 2σ(*I*)]^*a*^	0.1230	0.0343	0.0307	0.0334
*wR*_2_[*I* > 2σ(*I*)]^*b*^	0.2979	0.0681	0.0709	0.0769

Notes: (*a*) *R*_1_ = Σ||*F*_o_| − |*F*_c_||/Σ|*F*_o_|, (*b*) *wR*_2_= {Σ[*w*(F_o_^2^ − *F*_c_^2^)^2^]/Σ[*w*(*F*_o_^2^)^2^]}^1/2^.

**Table 2:** Selected bond lengths (Å) for complex **1**

Cu1—O1	1.929 (2)	Cu2—O3	1.929 (2)	Cu3—O5	1.935 (2)
Cu1—O2	1.949 (2)	Cu2—O4	1.939 (2)	Cu3—O6	1.952 (2)
Cu1—N9	1.898 (2)	Cu2—N1	1.902 (3)	Cu3—N3	1.910 (3)
Cu1—N10	2.016 (3)	Cu2—N2	1.985 (3)	Cu3—N4	2.010 (3)
Cu1—O18w	2.470 (3)	Cu2—O15_(NO_3_)_	2.469 (3)	Cu3—O19w	2.444 (2)
Cu4—O7	1.938 (2)	Cu5—O9	1.931 (2)	Gd1—O1	2.457 (2)
Cu4—O8	1.953 (2)	Cu5—O10	1.939 (2)	Gd1—O3	2.381 (2)
Cu4—N5	1.906 (2)	Cu5—N7	1.901 (2)	Gd1—O5	2.483 (2)
Cu4—N6	2.005 (2)	Cu5—N8	2.022 (2)	Gd1—O7	2.408 (2)
Cu4—O20w	2.401 (3)	Cu5—O24w	2.449 (2)	Gd1—O9	2.414 (2)
C11—O12	1.284 (9)	C11*B*—O12*B*	1.310 (10)	Gd1—O11w	2.359 (2)
C11—O13	1.307 (10)	C11*B*—O13*B*	1.307 (10)	Gd1—O12	2.317 (11)
C11—O14	1.252 (9)	C11*B*—O14*B*	1.253 (9)	Gd1—O13	2.288 (17)
Gd1—O12*B*	2.396 (10)	Gd1—O13*B*	2.388 (17)		

**Table 3:** Selected bond lengths (Å) for complex **2**

Cu1—O1	1.931 (3)	Cu2—O3	1.931 (3)	Cu3—O5	1.941 (3)
Cu1—O2	1.949 (3)	Cu2—O4	1.939 (3)	Cu3—O6	1.954 (3)
Cu1—N9	1.897 (3)	Cu2—N1	1.907 (4)	Cu3—N3	1.905 (4)
Cu1—N10	2.012 (4)	Cu2—N2	1.983 (4)	Cu3—N4	2.017 (4)
Cu1—O18w	2.476 (3)	Cu2—O15_(NO_3_)_	2.464 (3)	Cu3—O19w	2.430 (3)
Cu4—O7	1.936 (3)	Cu5—O9	1.932 (3)	Dy1—O1	2.453 (3)
Cu4—O8	1.956 (3)	Cu5—O10	1.941 (3)	Dy1—O3	2.382 (3)
Cu4—N5	1.904 (3)	Cu5—N7	1.899 (3)	Dy1—O5	2.469 (3)
Cu4—N6	2.005 (3)	Cu5—N8	2.021 (3)	Dy1—O7	2.412 (3)
Cu4—O20w	2.400 (3)	Cu5—O24w	2.440 (3)	Dy1—O9	2.410 (3)
C11—O12	1.275 (16)	C11*B*—O12*B*	1.295 (9)	Dy1—O11w	2.357 (3)
C11—O13	1.318 (16)	C11*B*—O13*B*	1.326 (8)	Dy1—O12	2.27 (2)
C11—O14	1.256 (15)	C11*B*—O14*B*	1.251 (8)	Dy1—O13	2.31 (3)
Dy1—O12*B*	2.380 (8)	Dy1—O13*B*	2.347 (12)		

**Table 4:** Selected bond lengths (Å) for complex **3**

Cu1—O1	1.930 (2)	Cu2—O3	1.926 (2)	Cu3—O5	1.939 (2)
Cu1—O2	1.948 (2)	Cu2—O4	1.940 (2)	Cu3—O6	1.949 (2)
Cu1—N9	1.897 (2)	Cu2—N1	1.898 (3)	Cu3—N3	1.908 (3)
Cu1—N10	2.013 (3)	Cu2–N2	1.987 (3)	Cu3—N4	2.010 (3)
Cu1—O18w	2.471 (3)	Cu2—O15_(NO_3_)_	2.463 (3)	Cu3—O19w	2.437 (3)
Cu4—O7	1.937 (2)	Cu5—O9	1.931 (2)	Ho1—O1	2.446 (2)
Cu4—O8	1.949 (2)	Cu5—O10	1.941 (2)	Ho1—O3	2.374 (2)
Cu4—N5	1.903 (3)	Cu5—N7	1.902 (3)	Ho1—O5	2.475 (2)
Cu4—N6	2.008 (3)	Cu5—N8	2.019 (3)	Ho1—O7	2.404 (2)
Cu4—O20w	2.405 (3)	Cu5—O24w	2.443 (3)	Ho1—O9	2.407 (2)
C11—O12	1.239 (11)	C11*B*—O12*B*	1.262 (10)	Ho1—O11w	2.358 (2)
C11—O13	1.305 (13)	C11*B*—O13*B*	1.309 (10)	Ho1—O12	2.304 (16)
C11—O14	1.283 (12)	C11*B*—O14*B*	1.261 (9)	Ho1—O13	2.30 (3)
Ho1—O12*B*	2.374 (12)	Ho1—O13*B*	2.35 (2)		

**Table 5:** Selected bond angles (°) for complex **1**

O1—Cu1—O2	85.28 (9)	O3—Cu2—O4	85.63 (9)	O5—Cu3—O6	85.44 (9)
N9—Cu1—O1	89.32 (9)	N1—Cu2—O3	90.48 (9)	N3—Cu3—O5	90.98 (10)
O2—Cu1—N10	100.27 (10)	O4—Cu2—N2	100.83 (10)	O6—Cu3—N4	99.85 (11)-
N9–Cu1—N10	83.70 (10)	N1—Cu2—N2	82.87 (11)	N3—Cu3—N4	82.70 (12)
O7—Cu4—O8	84.56 (8)	O9—Cu5—O10	85.57 (8)	O3—Gd1—O1	71.71 (7)
N5—Cu4—O7	91.39 (9)	N7—Cu5—O9	89.59 (9)	O3—Gd1—O5	72.08 (7)
O8—Cu4—N6	98.79 (9)	O10—Cu5—N8	100.42 (9)	O7—Gd1—O5	72.87 (7)
N5—Cu4—N6	83.88 (10)	N7—Cu5—N8	83.14 (10)	O7—Gd1—O9	71.27 (6)
O13—Gd1—O12	56.4 (3)	O13*B*–Gd1–O12*B*	54.1 (3)	O9—Gd1—O1	70.62 (7)

**Table 6:** Selected bond angles (°) for complex **2**

O1—Cu1—O2	85.19 (12)	O3—Cu2—O4	85.41 (12)	O5–Cu3—O6	85.39 (12)
O1—Cu1—N9	89.39 (13)	O3—Cu2—N1	90.66 (13)	O5—Cu3—N3	90.67 (13)
O2—Cu1—N10	100.37 (13)	O4—Cu2—N2	101.00 (14)	O6—Cu3—N4	100.11 (14)
N9—Cu1—N10	83.73 (14)	N1—Cu2—N2	82.65 (15)	N3—Cu3—N4	82.79 (15)
O7—Cu4—O8	84.56 (11)	O9—Cu5—O10	85.47 (11)	O1—Dy1—O3	71.77 (9)
O7—Cu4—N5	91.62 (13)	O9—Cu5—N7	89.53 (12)	O3—Dy1—O5	71.92 (9)
O8—Cu4—N6	98.55 (13)	O10—Cu5—N8	100.49 (13)	O5—Dy1—O7	72.60 (9)
N5—Cu4—N6	83.78 (14)	N7—Cu5—N8	83.23 (14)	O7—Dy1—O9	71.21 (9)
O12—Dy1—O13	57.1 (6)	O12*B*–Dy1—O13*B*	54.7 (2)	O9—Dy1—O1	70.83 (9)

**Table 7:** Selected bond angles (°) for complex **3**

O1—Cu1—O2	85.31 (9)	O3—Cu2—O4	85.63 (9)	O5—Cu3—O6	85.53 (9)
O1—Cu1—N9	89.22 (10)	O3—Cu2—N1	90.61 (10)	O5—Cu3—N3	90.80 (10)
O2—Cu1—N10	100.11 (11)	O4–Cu2—N2	100.93 (11)	O6—Cu3—N4	99.97 (11)
N9—Cu1—N10	83.88 (11)	N1—Cu2—N2	82.56 (12)	N3—Cu3—N4	82.62 (12)
O7—Cu4—O8	84.51 (9)	O9—Cu5—O10	85.63 (9)	O1—Ho1—O3	71.84 (7)
O7—Cu4—N5	91.47 (10)	O9—Cu5—N7	89.41 (10)	O3—Ho1—O5	72.17 (7)
O8—Cu4—N6	98.74 (10)	O10—Cu5—N8	100.38 (10)	O5—Ho1—O7	72.61 (7)
N5—Cu4—N6	83.87 (11)	N7—Cu5—N8	83.25 (11)	O7—Ho1—O9	71.34 (7)
O12—Ho1—O13	56.5 (4)	O12*B*—Ho1—O13*B*	53.9 (3)	O9—Ho1—O1	70.69 (7)

**Table 8:** Comparison of selected characteristics of **1**–**3** with the earlier reported Eu^III^ analogue (CCDC127569; Stemmler *et al.*, 1999 ▸)

	CCDC127569	Complex **1**	Complex **2**	Complex **3**
	EuCu_5_	GdCu_5_	DyCu_5_	HoCu_5_
Range of *Ln*···Cu separations (Å)	3.890 (2)–3.911 (3)	3.8699 (5)–3.9097 (5)	3.8715 (5)–3.9016 (6)	3.8670 (5)–3.9021 (5)
Range of Cu···Cu separations (Å)	4.575 (3)–4.589 (3)	4.5677 (7)–4.5846 (7)	4.5645 (7)–4.5797 (8)	4.5583 (7)–4.5808 (7)
Range of *Ln*—O_equat_ (Å)	2.406 (11)–2.493 (11)	2.381 (2)–2.484 (2)	2.382 (3)–2.469 (3)	2.374 (2)–2.475 (2)
Range of *Ln*—O_carbonate_ (Å)	2.369 (13)–2.392 (15)	2.288 (17)–2.396 (10)	2.27 (2)–2.380 (8)	2.30 (3)–2.374 (12)
Range of Cu—O_equat_ (Å)	1.901 (11)–1.972 (10)	1.929 (2)–1.953 (2)	1.931 (3)–1.956 (4)	1.929 (2)–1.953 (2)
Range of Cu—N_equat_ (Å)	1.886 (14)–2.022 (13)	1.898 (2)–2.022 (2)	1.898 (3)–2.022 (3)	1.898 (2)–2.022 (2)
Range of τ values (Addison *et al.*, 1984 ▸) for penta­coordinate Cu^II^ ions	0.00–0.20	0.01–0.20	0.01–0.20	0.02–0.20
*Ln* ^III^ coordination number	8	8	8	8
Average deviation of non-hydrogen atoms from Cu_5_ plane (Å)	0.179	0.188	0.183	0.186
Largest deviation among non-hydrogen atoms from Cu_5_ plane (Å)	0.605	0.606	0.602	0.597
Deviation of *Ln* ^III^ ion from Cu_5_ plane (Å)	0.351	0.337	0.354	0.330

**Table 9:** Continuous shape calculations for octa­coordinated *Ln* ^3+^ ions in **1**–**3** obtained using *Shape2.1* software (Casanova *et al.*, 2005 ▸)

	Complex **1**	Complex **2**	Complex **3**
**OP-8**	31.930	31.846	31.915
**HPY-8**	22.627	22.698	22.560
**HBPY-8**	16.081	16.114	16.307
**CU-8**	12.990	12.970	12.794
**SAPR-8**	3.769	3.759	3.770
***TDD-8***	1.805	1.743	1.763
**JGBF-8**	12.037	12.418	12.302
**JETBPY-8**	27.751	27.478	27.602

**Table 10:** Hydrogen-bond geometry (Å, °) for complex **1** [Chem scheme1]

*D*—H⋯*A*	*D*—H	H⋯*A*	*D*⋯*A*	*D*—H⋯*A*
O23—H23*A*⋯O16^i^	0.85 (2)	1.81 (2)	2.642 (10)	165 (10)
O23—H23*B*⋯O14	0.84 (2)	1.90 (2)	2.644 (10)	148 (5)
N2—H2*A*⋯O13^i^	0.91	2.17	3.00 (2)	151
N2—H2*A*⋯O13*B* ^i^	0.91	2.06	2.92 (2)	157
N2—H2*B*⋯O16	0.91	2.25	3.068 (4)	150
N4—H4*A*⋯O21	0.91	2.08	2.932 (4)	155
N4—H4*B*⋯O23^ii^	0.91	1.91	2.604 (8)	132
N6—H6*A*⋯O16^iii^	0.91	2.24	3.061 (4)	150
N6—H6*B*⋯N5^iv^	0.91	2.53	3.268 (4)	139
N6—H6*B*⋯O5^iv^	0.91	2.46	3.357 (4)	171
N8—H8*A*⋯O7^v^	0.91	2.51	3.299 (4)	145
N8—H8*A*⋯O20^v^	0.91	2.39	3.005 (4)	125
N8—H8*B*⋯O22^vi^	0.91	2.15	3.033 (4)	163
N10—H10*A*⋯O17^vii^	0.91	2.22	3.025 (4)	146
N10—H10*B*⋯O11^viii^	0.91	2.41	3.192 (4)	145
O11—H11*A*⋯O25	0.84 (2)	1.84 (2)	2.662 (3)	166 (4)
O11—H11*B*⋯O24	0.84 (2)	1.98 (2)	2.798 (3)	167 (4)
O18—H18*A*⋯O4^i^	0.83 (2)	1.99 (2)	2.813 (3)	169 (4)
O18—H18*B*⋯O13	0.83 (2)	2.07 (2)	2.886 (14)	170 (4)
O18—H18*B*⋯O13*B*	0.83 (2)	1.79 (2)	2.612 (14)	169 (5)
O19—H19*A*⋯O8^iv^	0.84 (2)	1.89 (2)	2.719 (3)	167 (4)
O19—H19*B*⋯O18^ii^	0.84 (2)	2.01 (2)	2.847 (3)	176 (4)
O20—H20*A*⋯O10^v^	0.85 (2)	1.96 (3)	2.750 (3)	156 (5)
O20—H20*B*⋯O14^ix^	0.85 (2)	2.17 (2)	2.997 (9)	165 (4)
O20—H20*B*⋯O14*B* ^ix^	0.85 (2)	2.24 (2)	3.075 (9)	169 (4)
O21—H21*A*⋯O6^x^	0.84 (2)	2.00 (2)	2.813 (3)	163 (5)
O21—H21*B*⋯O22	0.85 (2)	1.83 (2)	2.650 (5)	162 (5)
O22—H22*A*⋯O14^ix^	0.86 (2)	1.96 (4)	2.742 (9)	150 (6)
O22—H22*A*⋯O14*B* ^ix^	0.86 (2)	1.89 (3)	2.730 (8)	166 (6)
O22—H22*B*⋯O12	0.87 (2)	1.74 (3)	2.594 (12)	168 (6)
O22—H22*B*⋯O14	0.87 (2)	2.49 (5)	3.137 (9)	131 (5)
O22—H22*B*⋯O12*B*	0.87 (2)	1.94 (2)	2.803 (11)	176 (6)
O24—H24*A*⋯O2^viii^	0.83 (2)	1.98 (2)	2.785 (3)	163 (4)
O24—H24*B*⋯O21^vi^	0.84 (2)	1.95 (2)	2.755 (3)	161 (4)
O25—H25*A*⋯O19	0.86 (2)	1.92 (2)	2.773 (3)	173 (5)
O25—H25*B*⋯O15	0.85 (2)	1.93 (2)	2.754 (4)	166 (4)

Symmetry codes: (i) 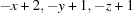; (ii) 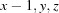; (iii) 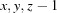; (iv) 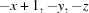; (v) 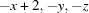; (vi) 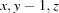; (vii) 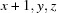; (viii) 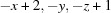; (ix) 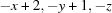; (x) 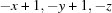.

**Table 11:** Hydrogen-bond geometry (Å, °) for complex **2** [Chem scheme1]

*D*—H⋯*A*	*D*—H	H⋯*A*	*D*⋯*A*	*D*—H⋯*A*
O23—H23*A*⋯O16^i^	0.84	1.84	2.61 (2)	150
O23—H23*B*⋯O14	0.84	1.95	2.657 (17)	141
N2—H2*A*⋯O13^i^	0.91	2.23	3.05 (3)	150
N2—H2*A*⋯O13*B* ^i^	0.91	2.05	2.900 (11)	156
N2—H2*B*⋯O16	0.91	2.23	3.054 (6)	150
N4—H4*A*⋯O21	0.91	2.08	2.912 (6)	152
N4—H4*B*⋯O23^ii^	0.91	1.86	2.520 (16)	128
N6—H6*A*⋯O16^iii^	0.91	2.29	3.091 (6)	147
N6—H6*B*⋯N5^iv^	0.91	2.54	3.287 (5)	140
N6—H6*B*⋯O5^iv^	0.91	2.49	3.396 (5)	171
N8—H8*A*⋯O7^v^	0.91	2.48	3.262 (5)	144
N8—H8*A*⋯O20^v^	0.91	2.38	2.989 (5)	124
N8—H8*B*⋯O22^vi^	0.91	2.17	3.045 (6)	162
N10—H10*A*⋯O17^vii^	0.91	2.20	3.005 (5)	147
N10—H10*B*⋯O11^viii^	0.91	2.42	3.198 (5)	143
O11—H11*A*⋯O25	0.83 (2)	1.86 (3)	2.642 (4)	157 (6)
O11—H11*B*⋯O24	0.82 (2)	2.00 (3)	2.800 (4)	164 (6)
O18—H18*A*⋯O4^i^	0.83 (2)	2.01 (2)	2.821 (4)	165 (6)
O18—H18*B*⋯O13	0.83 (2)	2.05 (4)	2.87 (3)	169 (6)
O18—H18*B*⋯O13*B*	0.83 (2)	1.84 (3)	2.650 (10)	165 (6)
O19—H19*A*⋯O8^iv^	0.82 (2)	1.91 (2)	2.710 (4)	167 (6)
O19—H19*B*⋯O18^ii^	0.82 (2)	2.01 (2)	2.827 (4)	172 (6)
O20—H20*A*⋯O10^v^	0.82 (2)	1.95 (3)	2.738 (4)	160 (6)
O20—H20*B*⋯O14^ix^	0.83 (2)	2.18 (3)	2.973 (18)	158 (6)
O20—H20*B*⋯O14*B* ^ix^	0.83 (2)	2.28 (3)	3.065 (7)	158 (6)
O21—H21*A*⋯O6^x^	0.84 (2)	1.98 (2)	2.810 (4)	174 (7)
O21—H21*B*⋯O22	0.84 (2)	1.85 (3)	2.660 (6)	160 (7)
O22—H22*A*⋯O14^ix^	0.85 (2)	1.91 (3)	2.736 (19)	166 (8)
O22—H22*A*⋯O14*B* ^ix^	0.85 (2)	1.87 (2)	2.708 (8)	171 (8)
O22—H22*B*⋯O12	0.85 (2)	1.74 (4)	2.57 (2)	165 (8)
O22—H22*B*⋯O14	0.85 (2)	2.41 (7)	3.033 (18)	130 (7)
O22—H22*B*⋯O12*B*	0.85 (2)	1.90 (2)	2.753 (10)	179 (9)
O24—H24*A*⋯O2^viii^	0.83 (2)	1.97 (2)	2.777 (4)	165 (5)
O24—H24*B*⋯O21^vi^	0.83 (2)	1.95 (2)	2.767 (5)	168 (5)
O25—H25*A*⋯O19	0.83 (2)	1.95 (2)	2.769 (5)	168 (6)
O25—H25*B*⋯O15	0.83 (2)	1.94 (3)	2.752 (5)	163 (6)

Symmetry codes: (i) 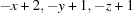; (ii) 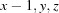; (iii) 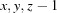; (iv) 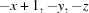; (v) 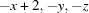; (vi) 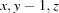; (vii) 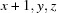; (viii) 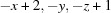; (ix) 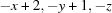; (x) 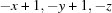.

**Table 12:** Hydrogen-bond geometry (Å, °) for complex **3** [Chem scheme1]

*D*—H⋯*A*	*D*—H	H⋯*A*	*D*⋯*A*	*D*—H⋯*A*
O23—H23*A*⋯O16^i^	0.88	1.87	2.747 (11)	173
O23—H23*B*⋯O14	0.83	1.98	2.509 (13)	121
N2—H2*A*⋯O13^i^	0.91	2.17	3.00 (3)	150
N2—H2*A*⋯O13*B* ^i^	0.91	2.05	2.90 (2)	155
N2—H2*B*⋯O16	0.91	2.26	3.078 (5)	149
N4—H4*A*⋯O21	0.91	2.07	2.914 (5)	154
N4—H4*B*⋯O23^ii^	0.91	1.98	2.726 (10)	138
N6—H6*A*⋯O16^iii^	0.91	2.25	3.060 (5)	149
N6—H6*B*⋯N5^iv^	0.91	2.52	3.267 (4)	139
N6—H6*B*⋯O5^iv^	0.91	2.46	3.359 (4)	171
N8—H8*A*⋯O7^v^	0.91	2.49	3.285 (4)	146
N8—H8*A*⋯O20^v^	0.91	2.41	3.017 (4)	124
N8—H8*B*⋯O22^vi^	0.91	2.17	3.048 (5)	163
N10—H10*A*⋯O17^vii^	0.91	2.23	3.024 (4)	145
N10—H10*B*⋯O11^viii^	0.91	2.39	3.183 (4)	146
O11—H11*A*⋯O25	0.82 (2)	1.86 (2)	2.660 (4)	165 (5)
O11—H11*B*⋯O24	0.81 (2)	2.00 (2)	2.801 (4)	166 (5)
O18—H18*A*⋯O4^i^	0.82 (2)	2.01 (3)	2.801 (3)	160 (5)
O18—H18*B*⋯O13	0.83 (2)	2.04 (3)	2.87 (2)	173 (5)
O18—H18*B*⋯O13*B*	0.83 (2)	1.84 (3)	2.671 (18)	171 (5)
O19—H19*A*⋯O8^iv^	0.82 (2)	1.90 (2)	2.713 (3)	174 (5)
O19—H19*B*⋯O18^ii^	0.83 (2)	2.02 (2)	2.840 (4)	174 (5)
O20—H20*A*⋯O10^v^	0.82 (2)	1.96 (3)	2.732 (3)	156 (5)
O20—H20*B*⋯O14^ix^	0.81 (2)	2.21 (3)	2.997 (12)	162 (5)
O20—H20*B*⋯O14*B* ^ix^	0.81 (2)	2.27 (3)	3.060 (9)	163 (5)
O21—H21*A*⋯O6^x^	0.82 (2)	2.07 (3)	2.813 (4)	151 (6)
O21—H21*B*⋯O22	0.83 (2)	1.87 (3)	2.638 (5)	154 (6)
O22—H22*A*⋯O14^ix^	0.87 (2)	1.88 (3)	2.736 (12)	170 (7)
O22—H22*A*⋯O14*B* ^ix^	0.87 (2)	1.84 (2)	2.709 (9)	173 (7)
O22—H22*B*⋯O12	0.86 (2)	1.74 (3)	2.600 (16)	170 (7)
O22—H22*B*⋯O14	0.86 (2)	2.51 (6)	3.088 (11)	125 (6)
O22—H22*B*⋯O12*B*	0.86 (2)	1.93 (3)	2.791 (14)	173 (7)
O24—H24*A*⋯O2^viii^	0.81 (2)	1.99 (2)	2.781 (3)	166 (5)
O24—H24*B*⋯O21^vi^	0.82 (2)	1.94 (2)	2.759 (4)	174 (5)
O25—H25*A*⋯O19	0.82 (2)	1.96 (2)	2.779 (4)	177 (5)
O25—H25*B*⋯O15	0.83 (2)	1.95 (2)	2.751 (4)	165 (5)

Symmetry codes: (i) 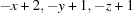; (ii) 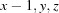; (iii) 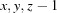; (iv) 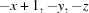; (v) 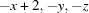; (vi) 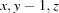; (vii) 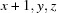; (viii) 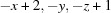; (ix) 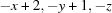; (x) 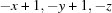.

**Table 13:** Experimental details

	Complex **1**	Complex **2**	Complex **3**
Crystal data			
Chemical formula	[Cu_5_Gd(C_2_H_4_N_2_O_2_)_5_(CO_3_)(NO_3_)(H_2_O)_5_]·3.5H_2_O	[Cu_5_Dy(C_2_H_4_N_2_O_2_)_5_(CO_3_)(NO_3_)(H_2_O)_5_]·3.28H_2_O	[Cu_5_Ho(C_2_H_4_N_2_O_2_)_5_(CO_3_)(NO_3_)(H_2_O)_5_]·3.445H_2_O
*M*_r_	1190.49	1191.77	1197.21
Crystal system, space group	Triclinic, *P* 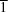	Triclinic, *P* 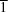	Triclinic, *P* 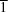
Temperature (K)	150	150	150
*a*, *b*, *c* (Å)	11.2057 (15), 11.5054 (15), 13.2983 (10)	11.1083 (5), 11.4991 (5), 13.2894 (6)	11.2027 (9), 11.4955 (9), 13.2467 (10)
α, β, γ (°)	94.026 (4), 94.942 (3), 107.558 (3)	93.9235 (16), 94.7713 (17), 107.1470 (17)	94.001 (3), 94.784 (3), 107.518 (3)
*V* (Å^3^)	1620.2 (3)	1608.73 (13)	1613.0 (2)
*Z*	2	2	2
Radiation type	Mo *K*α	Mo *K*α	Mo *K*α
μ (mm^−1^)	5.35	5.65	5.77
Crystal size (mm)	0.15 × 0.10 × 0.05	0.25 × 0.21 × 0.11	0.44 × 0.42 × 0.28

Data collection			
Diffractometer	Bruker AXS D8 Quest CMOS	Bruker AXS D8 Quest CMOS	Bruker AXS D8 Quest CMOS
Absorption correction	Multi-scan (*SADABS*; Krause *et al.*, 2015 ▸)	Multi-scan (*SADABS*; Krause *et al.*, 2015 ▸)	Multi-scan (*SADABS*; Krause *et al.*, 2015 ▸)
*T*_min_, *T*_max_	0.599, 0.747	0.648, 0.754	0.548, 0.747
No. of measured, independent and observed [*I* > 2σ(*I*)] reflections	118155, 12399, 9545	74286, 8274, 6997	50443, 12306, 10012
*R*_int_	0.056	0.048	0.042
(sin θ/λ)_max_ (Å^−1^)	0.771	0.676	0.771

Refinement			
*R*[*F* ^2^ > 2σ(*F* ^2^)], *wR*(*F* ^2^), *S*	0.034, 0.076, 1.05	0.031, 0.076, 1.08	0.033, 0.082, 1.05
No. of reflections	12399	8274	12306
No. of parameters	570	564	563
No. of restraints	135	133	139
H-atom treatment	H atoms treated by a mixture of independent and constrained refinement	H atoms treated by a mixture of independent and constrained refinement	H atoms treated by a mixture of independent and constrained refinement
Δρ_max_, Δρ_min_ (e Å^−3^)	2.06, −1.40	0.99, −1.26	2.80, −2.27

Computer programs: *APEX3* and *SAINT* (Bruker, 2017 ▸), *SHELXS97* (Sheldrick, 2008 ▸), *SHELXL2018/3* (Sheldrick, 2015 ▸), *SHELXLE* (Hübschle *et al.*, 2011 ▸) and *publCIF* (Westrip, 2010 ▸).

## supplementary crystallographic information

### Pentaaquacarbonatopentakis(glycine hydroxamato)nitratopentacopper(II)gadolinium(III) 3.5-hydrate (Complex_1) Crystal data

**Table:**

[Cu_5_Gd(C_2_H_4_N_2_O_2_)_5_(CO_3_)(NO_3_)(H_2_O)_5_]·3.5H_2_O	*Z* = 2
*M**_r_* = 1190.49	*F*(000) = 1170
Triclinic, *P*1	*D*_x_ = 2.440 Mg m^−^^3^
*a* = 11.2057 (15) Å	Mo *K*α radiation, λ = 0.71073 Å
*b* = 11.5054 (15) Å	Cell parameters from 9949 reflections
*c* = 13.2983 (10) Å	θ = 3.1–32.5°
α = 94.026 (4)°	µ = 5.35 mm^−^^1^
β = 94.942 (3)°	*T* = 150 K
γ = 107.558 (3)°	Rod, blue
*V* = 1620.2 (3) Å^3^	0.15 × 0.10 × 0.05 mm

### Pentaaquacarbonatopentakis(glycine hydroxamato)nitratopentacopper(II)gadolinium(III) 3.5-hydrate (Complex_1) Data collection

**Table:**

Bruker AXS D8 Quest CMOS diffractometer	12399 independent reflections
Radiation source: sealed tube X-ray source	9545 reflections with *I* > 2σ(*I*)
Triumph curved graphite crystal monochromator	*R*_int_ = 0.056
ω and phi scans	θ_max_ = 33.2°, θ_min_ = 3.1°
Absorption correction: multi-scan (SADABS; Krause *et al.*, 2015)	*h* = −17→17
*T*_min_ = 0.599, *T*_max_ = 0.747	*k* = −17→17
118155 measured reflections	*l* = −19→20

### Pentaaquacarbonatopentakis(glycine hydroxamato)nitratopentacopper(II)gadolinium(III) 3.5-hydrate (Complex_1) Refinement

**Table:**

Refinement on *F*^2^	Secondary atom site location: difference Fourier map
Least-squares matrix: full	Hydrogen site location: mixed
*R*[*F*^2^ > 2σ(*F*^2^)] = 0.034	H atoms treated by a mixture of independent and constrained refinement
*wR*(*F*^2^) = 0.076	*w* = 1/[σ^2^(*F*_o_^2^) + (0.0346*P*)^2^ + 2.5135*P*] where *P* = (*F*_o_^2^ + 2*F*_c_^2^)/3
*S* = 1.05	(Δ/σ)_max_ = 0.001
12399 reflections	Δρ_max_ = 2.06 e Å^−^^3^
570 parameters	Δρ_min_ = −1.40 e Å^−^^3^
135 restraints	Extinction correction: SHELXL-2018/3 (Sheldrick 2015), Fc^*^=kFc[1+0.001xFc^2^λ^3^/sin(2θ)]^-1/4^
Primary atom site location: structure-invariant direct methods	Extinction coefficient: 0.00141 (15)

### Pentaaquacarbonatopentakis(glycine hydroxamato)nitratopentacopper(II)gadolinium(III) 3.5-hydrate (Complex_1) Special details

**Table:**

Geometry. All esds (except the esd in the dihedral angle between two l.s. planes) are estimated using the full covariance matrix. The cell esds are taken into account individually in the estimation of esds in distances, angles and torsion angles; correlations between esds in cell parameters are only used when they are defined by crystal symmetry. An approximate (isotropic) treatment of cell esds is used for estimating esds involving l.s. planes.
Refinement. A water molecule is partially occupied, inducing disorder for the nearby carbonate anion. The two disordered moieties were restrained to have similar geometries. Uij components of ADPs for disordered atoms closer to each other than 2.0 Angstrom were restrained to be similar. The distance of the water oxygen to one of the carbonate oxygen atoms was restrained to be at least 2.8 Angstrom for the moiety that contains the water molecule. Water H atom positions were refined and O-H and H···H distances were restrained to 0.84 (2) and 1.36 (2) Angstrom, respectively. The water H atom positions of the disordered moiety were further restrained based on hydrogen bonding considerations. Subject to these conditions the occupancy ratio refined to 0.499 (11) to 0.501 (11).

### Pentaaquacarbonatopentakis(glycine hydroxamato)nitratopentacopper(II)gadolinium(III) 3.5-hydrate (Complex_1) Fractional atomic coordinates and isotropic or equivalent isotropic displacement parameters (Å^2^)

**Table:**

	*x*	*y*	*z*	*U*_iso_*/*U*_eq_	Occ. (<1)
C1	1.0282 (3)	0.3071 (3)	0.5735 (2)	0.0169 (5)	
C2	1.0133 (3)	0.3907 (3)	0.6602 (2)	0.0198 (6)	
H2C	1.011450	0.350005	0.723478	0.024*	
H2D	1.085802	0.466751	0.669686	0.024*	
C3	0.6601 (3)	0.4525 (3)	0.3876 (2)	0.0184 (5)	
C4	0.5624 (3)	0.5110 (3)	0.3531 (2)	0.0237 (6)	
H4C	0.492326	0.489577	0.395853	0.028*	
H4D	0.600253	0.601279	0.361189	0.028*	
C5	0.5301 (3)	0.2146 (3)	0.0120 (2)	0.0180 (5)	
C6	0.4908 (3)	0.1751 (3)	−0.0995 (2)	0.0211 (6)	
H6C	0.398256	0.136861	−0.110988	0.025*	
H6D	0.513050	0.247914	−0.137828	0.025*	
C7	0.8131 (3)	−0.0807 (3)	−0.0320 (2)	0.0158 (5)	
C8	0.8535 (3)	−0.1924 (3)	−0.0560 (2)	0.0185 (5)	
H8C	0.778171	−0.265599	−0.071359	0.022*	
H8D	0.899628	−0.182227	−0.116646	0.022*	
C9	1.1071 (3)	−0.0372 (3)	0.3184 (2)	0.0155 (5)	
C10	1.2060 (3)	−0.0372 (3)	0.4025 (2)	0.0182 (5)	
H10C	1.183398	−0.118107	0.429579	0.022*	
H10D	1.288131	−0.023541	0.375327	0.022*	
C11	1.0002 (9)	0.3923 (10)	0.1657 (8)	0.0187 (17)	0.499 (11)
O12	0.8812 (8)	0.3500 (10)	0.1377 (9)	0.0268 (16)	0.499 (11)
O13	1.0398 (17)	0.3359 (17)	0.2371 (11)	0.0221 (18)	0.499 (11)
O14	1.0726 (7)	0.4812 (8)	0.1298 (7)	0.0296 (16)	0.499 (11)
O23	1.3130 (8)	0.5342 (7)	0.1998 (11)	0.100 (5)	0.499 (11)
H23A	1.324 (17)	0.608 (5)	0.223 (7)	0.150*	0.499 (11)
H23B	1.242 (5)	0.491 (7)	0.171 (13)	0.150*	0.499 (11)
C11B	1.0371 (9)	0.3913 (10)	0.1761 (9)	0.0210 (18)	0.501 (11)
O12B	0.9211 (8)	0.3493 (10)	0.1322 (8)	0.0280 (16)	0.501 (11)
O13B	1.0555 (17)	0.3350 (16)	0.2552 (11)	0.0231 (19)	0.501 (11)
O14B	1.1203 (8)	0.4785 (8)	0.1474 (6)	0.0319 (18)	0.501 (11)
N1	0.9476 (2)	0.2923 (2)	0.49334 (18)	0.0184 (5)	
N2	0.8943 (3)	0.4211 (3)	0.6387 (2)	0.0242 (5)	
H2A	0.909529	0.503064	0.653694	0.029*	
H2B	0.836962	0.381155	0.679007	0.029*	
N3	0.6792 (3)	0.3745 (2)	0.32041 (19)	0.0194 (5)	
N4	0.5128 (3)	0.4690 (3)	0.2453 (2)	0.0321 (7)	
H4A	0.541406	0.531489	0.206441	0.038*	
H4B	0.427124	0.446800	0.238524	0.038*	
N5	0.6111 (2)	0.1674 (2)	0.05484 (18)	0.0177 (5)	
N6	0.5532 (2)	0.0865 (2)	−0.13735 (18)	0.0190 (5)	
H6A	0.585643	0.108822	−0.196056	0.023*	
H6B	0.496054	0.010374	−0.150377	0.023*	
N7	0.8504 (2)	−0.0252 (2)	0.05833 (18)	0.0161 (4)	
N8	0.9361 (2)	−0.2106 (2)	0.03158 (19)	0.0193 (5)	
H8A	1.014048	−0.203019	0.012836	0.023*	
H8B	0.903608	−0.287467	0.050144	0.023*	
N9	1.0599 (2)	0.0527 (2)	0.32669 (17)	0.0157 (4)	
N10	1.2174 (3)	0.0598 (3)	0.4855 (2)	0.0236 (5)	
H10A	1.297991	0.111081	0.495577	0.028*	
H10B	1.199114	0.025111	0.543971	0.028*	
N11	0.5782 (3)	0.2254 (3)	0.6118 (2)	0.0282 (6)	
Cu1	1.09871 (3)	0.15598 (3)	0.45052 (3)	0.01629 (7)	
Cu2	0.82345 (3)	0.37425 (3)	0.49429 (3)	0.01666 (7)	
Cu3	0.56789 (4)	0.32576 (3)	0.19730 (3)	0.01924 (8)	
Cu4	0.69162 (3)	0.08373 (3)	−0.03230 (3)	0.01653 (7)	
Cu5	0.95028 (3)	−0.08613 (3)	0.15079 (3)	0.01552 (7)	
Gd1	0.84770 (2)	0.19381 (2)	0.24301 (2)	0.01278 (4)	
O1	0.9539 (2)	0.2085 (2)	0.41509 (15)	0.0205 (4)	
O2	1.1140 (2)	0.2528 (2)	0.57995 (15)	0.0193 (4)	
O3	0.7698 (2)	0.31983 (19)	0.35249 (15)	0.0186 (4)	
O4	0.7179 (2)	0.47939 (19)	0.47886 (16)	0.0204 (4)	
O5	0.6439 (2)	0.2014 (2)	0.15887 (15)	0.0208 (4)	
O6	0.4844 (2)	0.2916 (2)	0.05861 (16)	0.0213 (4)	
O7	0.8123 (2)	0.07808 (19)	0.07923 (15)	0.0188 (4)	
O8	0.7441 (2)	−0.04598 (19)	−0.09994 (15)	0.0184 (4)	
O9	0.97139 (19)	0.05463 (18)	0.24655 (15)	0.0164 (4)	
O10	1.0746 (2)	−0.12244 (19)	0.24335 (15)	0.0178 (4)	
O11	0.7121 (2)	0.0139 (2)	0.29536 (17)	0.0213 (4)	
H11A	0.643 (3)	0.014 (4)	0.316 (3)	0.032*	
H11B	0.719 (4)	−0.056 (2)	0.281 (3)	0.032*	
O15	0.6328 (2)	0.2178 (2)	0.53415 (19)	0.0310 (5)	
O16	0.6467 (3)	0.2508 (4)	0.6961 (2)	0.0585 (10)	
O17	0.4642 (3)	0.2055 (3)	0.6078 (3)	0.0426 (7)	
O18	1.2479 (2)	0.3217 (2)	0.37422 (18)	0.0241 (5)	
H18A	1.266 (4)	0.378 (3)	0.421 (2)	0.036*	
H18B	1.192 (3)	0.336 (4)	0.336 (3)	0.036*	
O19	0.3869 (2)	0.1932 (2)	0.26751 (17)	0.0235 (5)	
H19A	0.338 (3)	0.154 (4)	0.216 (2)	0.035*	
H19B	0.348 (4)	0.234 (3)	0.297 (3)	0.035*	
O20	0.8216 (2)	0.2497 (2)	−0.11207 (19)	0.0283 (5)	
H20A	0.844 (4)	0.221 (4)	−0.165 (2)	0.042*	
H20B	0.839 (4)	0.3266 (19)	−0.113 (4)	0.042*	
O21	0.6426 (3)	0.6180 (2)	0.0939 (2)	0.0357 (6)	
H21A	0.599 (4)	0.629 (5)	0.043 (3)	0.054*	
H21B	0.685 (4)	0.572 (4)	0.075 (4)	0.054*	
O22	0.8172 (4)	0.5178 (3)	0.0465 (3)	0.0538 (9)	
H22A	0.824 (6)	0.514 (6)	−0.0174 (18)	0.081*	
H22B	0.850 (6)	0.468 (5)	0.076 (4)	0.081*	
O24	0.7763 (2)	−0.1972 (2)	0.24123 (17)	0.0213 (4)	
H24A	0.805 (4)	−0.228 (4)	0.289 (2)	0.032*	
H24B	0.739 (4)	−0.264 (2)	0.206 (3)	0.032*	
O25	0.5128 (2)	0.0541 (2)	0.36852 (19)	0.0266 (5)	
H25A	0.477 (4)	0.102 (4)	0.340 (3)	0.040*	
H25B	0.541 (4)	0.095 (4)	0.425 (2)	0.040*	

### Pentaaquacarbonatopentakis(glycine hydroxamato)nitratopentacopper(II)gadolinium(III) 3.5-hydrate (Complex_1) Atomic displacement parameters (Å^2^)

**Table:**

	*U*^11^	*U*^22^	*U*^33^	*U*^12^	*U*^13^	*U*^23^
C1	0.0204 (13)	0.0140 (12)	0.0141 (12)	0.0016 (10)	0.0034 (10)	0.0020 (9)
C2	0.0288 (15)	0.0167 (13)	0.0115 (12)	0.0042 (11)	0.0009 (11)	−0.0007 (10)
C3	0.0241 (14)	0.0139 (12)	0.0158 (12)	0.0040 (11)	0.0028 (11)	−0.0003 (10)
C4	0.0318 (17)	0.0204 (14)	0.0204 (14)	0.0120 (13)	0.0012 (12)	−0.0030 (11)
C5	0.0200 (13)	0.0194 (13)	0.0147 (12)	0.0062 (11)	0.0016 (10)	0.0022 (10)
C6	0.0246 (15)	0.0286 (15)	0.0134 (12)	0.0143 (12)	−0.0004 (11)	−0.0001 (11)
C7	0.0177 (13)	0.0158 (12)	0.0133 (12)	0.0046 (10)	0.0019 (10)	0.0000 (9)
C8	0.0252 (14)	0.0177 (13)	0.0133 (12)	0.0089 (11)	0.0009 (10)	−0.0027 (10)
C9	0.0164 (12)	0.0152 (12)	0.0142 (12)	0.0040 (10)	0.0010 (10)	0.0024 (9)
C10	0.0190 (13)	0.0186 (13)	0.0181 (13)	0.0075 (11)	−0.0003 (10)	0.0037 (10)
C11	0.019 (4)	0.021 (3)	0.016 (3)	0.006 (3)	0.003 (3)	−0.002 (2)
O12	0.019 (4)	0.027 (2)	0.030 (3)	0.000 (3)	0.002 (3)	0.011 (2)
O13	0.021 (4)	0.023 (3)	0.019 (4)	0.003 (3)	−0.001 (3)	0.005 (3)
O14	0.027 (4)	0.027 (3)	0.028 (3)	−0.003 (3)	0.003 (3)	0.006 (2)
O23	0.047 (5)	0.050 (5)	0.203 (13)	0.028 (4)	−0.018 (6)	−0.007 (6)
C11B	0.024 (4)	0.019 (2)	0.017 (3)	0.005 (3)	0.002 (3)	−0.005 (2)
O12B	0.026 (4)	0.027 (2)	0.024 (3)	−0.003 (3)	0.001 (3)	0.011 (2)
O13B	0.022 (4)	0.020 (3)	0.023 (5)	0.001 (3)	−0.005 (3)	0.001 (3)
O14B	0.036 (4)	0.026 (3)	0.020 (3)	−0.010 (3)	0.009 (3)	−0.002 (2)
N1	0.0251 (13)	0.0185 (11)	0.0115 (10)	0.0072 (10)	0.0040 (9)	−0.0032 (9)
N2	0.0282 (14)	0.0270 (13)	0.0164 (12)	0.0092 (11)	−0.0003 (10)	−0.0054 (10)
N3	0.0264 (13)	0.0174 (11)	0.0168 (11)	0.0109 (10)	0.0008 (10)	0.0010 (9)
N4	0.056 (2)	0.0267 (14)	0.0207 (13)	0.0252 (14)	−0.0001 (13)	0.0008 (11)
N5	0.0210 (12)	0.0238 (12)	0.0102 (10)	0.0106 (10)	−0.0005 (9)	−0.0003 (9)
N6	0.0215 (12)	0.0231 (12)	0.0123 (10)	0.0076 (10)	0.0003 (9)	−0.0002 (9)
N7	0.0213 (12)	0.0164 (11)	0.0131 (10)	0.0106 (9)	−0.0004 (9)	−0.0011 (8)
N8	0.0221 (12)	0.0195 (12)	0.0177 (11)	0.0098 (10)	−0.0006 (9)	−0.0011 (9)
N9	0.0177 (11)	0.0159 (11)	0.0130 (10)	0.0054 (9)	−0.0021 (8)	0.0007 (8)
N10	0.0225 (13)	0.0275 (13)	0.0199 (12)	0.0088 (11)	−0.0050 (10)	0.0003 (10)
N11	0.0271 (14)	0.0270 (14)	0.0263 (14)	0.0020 (11)	0.0040 (11)	0.0032 (11)
Cu1	0.01724 (16)	0.01941 (16)	0.01143 (15)	0.00596 (13)	−0.00150 (12)	−0.00123 (12)
Cu2	0.02216 (18)	0.01550 (16)	0.01149 (15)	0.00558 (13)	0.00110 (13)	−0.00207 (12)
Cu3	0.02855 (19)	0.01987 (17)	0.01282 (15)	0.01423 (15)	−0.00091 (14)	−0.00107 (13)
Cu4	0.01952 (17)	0.02133 (17)	0.01026 (15)	0.01014 (14)	−0.00125 (12)	−0.00179 (12)
Cu5	0.01973 (17)	0.01519 (15)	0.01250 (15)	0.00790 (13)	−0.00066 (12)	−0.00106 (12)
Gd1	0.01725 (7)	0.01235 (6)	0.00865 (6)	0.00532 (5)	−0.00028 (4)	−0.00060 (4)
O1	0.0234 (11)	0.0277 (11)	0.0114 (9)	0.0128 (9)	−0.0023 (8)	−0.0074 (8)
O2	0.0218 (10)	0.0217 (10)	0.0135 (9)	0.0064 (8)	−0.0011 (8)	0.0008 (8)
O3	0.0256 (11)	0.0203 (10)	0.0129 (9)	0.0124 (8)	0.0000 (8)	0.0001 (7)
O4	0.0254 (11)	0.0190 (10)	0.0159 (9)	0.0065 (8)	0.0025 (8)	−0.0031 (8)
O5	0.0281 (11)	0.0277 (11)	0.0102 (9)	0.0170 (9)	−0.0026 (8)	−0.0049 (8)
O6	0.0283 (11)	0.0263 (11)	0.0142 (9)	0.0169 (9)	−0.0010 (8)	0.0004 (8)
O7	0.0251 (11)	0.0193 (10)	0.0150 (9)	0.0139 (8)	−0.0021 (8)	−0.0036 (7)
O8	0.0233 (10)	0.0213 (10)	0.0119 (9)	0.0107 (8)	−0.0013 (8)	−0.0025 (7)
O9	0.0198 (10)	0.0170 (9)	0.0125 (9)	0.0083 (8)	−0.0047 (7)	−0.0016 (7)
O10	0.0209 (10)	0.0176 (9)	0.0163 (9)	0.0090 (8)	0.0005 (8)	0.0003 (7)
O11	0.0194 (10)	0.0185 (10)	0.0254 (11)	0.0043 (8)	0.0041 (9)	0.0024 (8)
O15	0.0318 (13)	0.0314 (13)	0.0280 (12)	0.0052 (10)	0.0081 (10)	0.0059 (10)
O16	0.0445 (18)	0.087 (3)	0.0234 (14)	−0.0098 (17)	0.0007 (13)	0.0051 (15)
O17	0.0271 (13)	0.0321 (14)	0.070 (2)	0.0112 (11)	0.0091 (13)	−0.0005 (14)
O18	0.0274 (12)	0.0211 (11)	0.0230 (11)	0.0090 (9)	−0.0002 (9)	−0.0060 (9)
O19	0.0230 (11)	0.0295 (12)	0.0177 (10)	0.0101 (9)	−0.0006 (8)	−0.0041 (9)
O20	0.0317 (13)	0.0248 (12)	0.0303 (13)	0.0103 (10)	0.0089 (10)	0.0017 (10)
O21	0.0499 (17)	0.0296 (13)	0.0284 (13)	0.0187 (12)	−0.0119 (12)	0.0000 (10)
O22	0.091 (3)	0.0470 (18)	0.0389 (17)	0.0372 (18)	0.0265 (18)	0.0148 (15)
O24	0.0249 (11)	0.0196 (10)	0.0181 (10)	0.0060 (9)	−0.0014 (8)	0.0027 (8)
O25	0.0262 (12)	0.0255 (12)	0.0279 (12)	0.0083 (10)	0.0031 (10)	0.0008 (9)

### Pentaaquacarbonatopentakis(glycine hydroxamato)nitratopentacopper(II)gadolinium(III) 3.5-hydrate (Complex_1) Geometric parameters (Å, º)

**Table:**

C1—O2	1.296 (4)	N5—Cu4	1.906 (2)
C1—N1	1.303 (4)	N6—Cu4	2.005 (2)
C1—C2	1.501 (4)	N6—H6A	0.9100
C2—N2	1.488 (4)	N6—H6B	0.9100
C2—H2C	0.9900	N7—O7	1.397 (3)
C2—H2D	0.9900	N7—Cu5	1.901 (2)
C3—O4	1.295 (4)	N8—Cu5	2.022 (2)
C3—N3	1.298 (4)	N8—H8A	0.9100
C3—C4	1.505 (4)	N8—H8B	0.9100
C4—N4	1.483 (4)	N9—O9	1.398 (3)
C4—H4C	0.9900	N9—Cu1	1.898 (2)
C4—H4D	0.9900	N10—Cu1	2.016 (3)
C5—O6	1.298 (4)	N10—H10A	0.9100
C5—N5	1.302 (4)	N10—H10B	0.9100
C5—C6	1.506 (4)	N11—O17	1.223 (4)
C6—N6	1.485 (4)	N11—O15	1.255 (4)
C6—H6C	0.9900	N11—O16	1.266 (4)
C6—H6D	0.9900	Cu1—O1	1.929 (2)
C7—N7	1.292 (4)	Cu1—O2	1.949 (2)
C7—O8	1.301 (3)	Cu1—O18	2.470 (3)
C7—C8	1.509 (4)	Cu2—O3	1.929 (2)
C8—N8	1.490 (4)	Cu2—O4	1.939 (2)
C8—H8C	0.9900	Cu2—O15	2.469 (3)
C8—H8D	0.9900	Cu3—O5	1.935 (2)
C9—O10	1.294 (3)	Cu3—O6	1.952 (2)
C9—N9	1.298 (4)	Cu3—O19	2.444 (2)
C9—C10	1.506 (4)	Cu4—O7	1.938 (2)
C10—N10	1.482 (4)	Cu4—O8	1.953 (2)
C10—H10C	0.9900	Cu4—O20	2.401 (3)
C10—H10D	0.9900	Cu5—O9	1.931 (2)
C11—O14	1.252 (9)	Cu5—O10	1.939 (2)
C11—O12	1.284 (9)	Cu5—O24	2.449 (2)
C11—O13	1.307 (10)	Gd1—O11	2.359 (2)
C11—Gd1	2.733 (10)	Gd1—O3	2.381 (2)
O12—Gd1	2.317 (11)	Gd1—O7	2.408 (2)
O13—Gd1	2.288 (17)	Gd1—O9	2.414 (2)
O23—H23A	0.851 (19)	Gd1—O1	2.457 (2)
O23—H23B	0.84 (2)	Gd1—O5	2.483 (2)
C11B—O14B	1.253 (9)	O11—H11A	0.840 (19)
C11B—O13B	1.307 (10)	O11—H11B	0.838 (19)
C11B—O12B	1.310 (10)	O18—H18A	0.829 (19)
C11B—Gd1	2.857 (10)	O18—H18B	0.830 (19)
O12B—Gd1	2.396 (10)	O19—H19A	0.844 (19)
O13B—Gd1	2.388 (17)	O19—H19B	0.836 (19)
N1—O1	1.389 (3)	O20—H20A	0.847 (19)
N1—Cu2	1.902 (3)	O20—H20B	0.849 (19)
N2—Cu2	1.985 (3)	O21—H21A	0.840 (19)
N2—H2A	0.9100	O21—H21B	0.849 (19)
N2—H2B	0.9100	O22—H22A	0.86 (2)
N3—O3	1.399 (3)	O22—H22B	0.87 (2)
N3—Cu3	1.910 (3)	O24—H24A	0.833 (19)
N4—Cu3	2.010 (3)	O24—H24B	0.839 (19)
N4—H4A	0.9100	O25—H25A	0.861 (19)
N4—H4B	0.9100	O25—H25B	0.845 (19)
N5—O5	1.397 (3)		
			
O2—C1—N1	123.2 (3)	O4—Cu2—O15	86.11 (9)
O2—C1—C2	121.7 (3)	N2—Cu2—O15	93.88 (11)
N1—C1—C2	115.1 (3)	N3—Cu3—O5	90.98 (10)
N2—C2—C1	109.7 (2)	N3—Cu3—O6	168.46 (11)
N2—C2—H2C	109.7	O5—Cu3—O6	85.44 (9)
C1—C2—H2C	109.7	N3—Cu3—N4	82.70 (12)
N2—C2—H2D	109.7	O5—Cu3—N4	172.15 (13)
C1—C2—H2D	109.7	O6—Cu3—N4	99.85 (11)
H2C—C2—H2D	108.2	N3—Cu3—O19	97.56 (10)
O4—C3—N3	124.2 (3)	O5—Cu3—O19	97.57 (9)
O4—C3—C4	120.7 (3)	O6—Cu3—O19	93.78 (9)
N3—C3—C4	115.1 (3)	N4—Cu3—O19	87.90 (12)
N4—C4—C3	110.7 (2)	N5—Cu4—O7	91.39 (9)
N4—C4—H4C	109.5	N5—Cu4—O8	161.67 (10)
C3—C4—H4C	109.5	O7—Cu4—O8	84.56 (8)
N4—C4—H4D	109.5	N5—Cu4—N6	83.88 (10)
C3—C4—H4D	109.5	O7—Cu4—N6	174.00 (10)
H4C—C4—H4D	108.1	O8—Cu4—N6	98.79 (9)
O6—C5—N5	124.4 (3)	N5—Cu4—O20	101.61 (10)
O6—C5—C6	120.0 (3)	O7—Cu4—O20	99.20 (9)
N5—C5—C6	115.6 (3)	O8—Cu4—O20	96.69 (9)
N6—C6—C5	111.2 (2)	N6—Cu4—O20	85.40 (10)
N6—C6—H6C	109.4	N7—Cu5—O9	89.59 (9)
C5—C6—H6C	109.4	N7—Cu5—O10	170.23 (10)
N6—C6—H6D	109.4	O9—Cu5—O10	85.57 (8)
C5—C6—H6D	109.4	N7—Cu5—N8	83.14 (10)
H6C—C6—H6D	108.0	O9—Cu5—N8	169.38 (10)
N7—C7—O8	123.8 (3)	O10—Cu5—N8	100.42 (9)
N7—C7—C8	116.0 (2)	N7—Cu5—O24	95.81 (9)
O8—C7—C8	120.2 (2)	O9—Cu5—O24	87.44 (8)
N8—C8—C7	110.4 (2)	O10—Cu5—O24	92.45 (8)
N8—C8—H8C	109.6	N8—Cu5—O24	100.96 (9)
C7—C8—H8C	109.6	O13—Gd1—O12	56.4 (3)
N8—C8—H8D	109.6	O13—Gd1—O11	153.9 (3)
C7—C8—H8D	109.6	O12—Gd1—O11	149.4 (2)
H8C—C8—H8D	108.1	O13—Gd1—O3	96.0 (6)
O10—C9—N9	123.9 (3)	O12—Gd1—O3	86.3 (3)
O10—C9—C10	120.3 (2)	O11—Gd1—O3	91.62 (8)
N9—C9—C10	115.8 (2)	O11—Gd1—O13B	147.7 (3)
N10—C10—C9	110.9 (2)	O3—Gd1—O13B	95.0 (5)
N10—C10—H10C	109.5	O11—Gd1—O12B	156.6 (2)
C9—C10—H10C	109.5	O3—Gd1—O12B	94.1 (3)
N10—C10—H10D	109.5	O13B—Gd1—O12B	54.1 (3)
C9—C10—H10D	109.5	O13—Gd1—O7	102.1 (5)
H10C—C10—H10D	108.0	O12—Gd1—O7	79.3 (3)
O14—C11—O12	123.6 (9)	O11—Gd1—O7	85.23 (8)
O14—C11—O13	122.2 (9)	O3—Gd1—O7	144.85 (7)
O12—C11—O13	114.2 (9)	O13B—Gd1—O7	106.2 (5)
O14—C11—Gd1	178.1 (8)	O12B—Gd1—O7	77.1 (3)
O12—C11—Gd1	57.7 (6)	O13—Gd1—O9	82.7 (5)
O13—C11—Gd1	56.5 (7)	O12—Gd1—O9	122.3 (2)
C11—O12—Gd1	94.3 (7)	O11—Gd1—O9	75.95 (7)
C11—O13—Gd1	95.0 (8)	O3—Gd1—O9	141.57 (7)
H23A—O23—H23B	119 (10)	O13B—Gd1—O9	79.4 (5)
O14B—C11B—O13B	123.7 (10)	O12B—Gd1—O9	111.8 (3)
O14B—C11B—O12B	123.8 (9)	O7—Gd1—O9	71.27 (6)
O13B—C11B—O12B	112.5 (9)	O13—Gd1—O1	76.8 (3)
O14B—C11B—Gd1	179.5 (9)	O12—Gd1—O1	125.7 (3)
O13B—C11B—Gd1	56.1 (7)	O11—Gd1—O1	81.98 (8)
O12B—C11B—Gd1	56.4 (5)	O3—Gd1—O1	71.71 (7)
C11B—O12B—Gd1	96.5 (7)	O13B—Gd1—O1	70.3 (3)
C11B—O13B—Gd1	96.9 (8)	O12B—Gd1—O1	121.3 (2)
C1—N1—O1	116.0 (2)	O7—Gd1—O1	141.68 (7)
C1—N1—Cu2	119.25 (19)	O9—Gd1—O1	70.62 (7)
O1—N1—Cu2	124.58 (18)	O13—Gd1—O5	125.4 (3)
C2—N2—Cu2	111.49 (18)	O12—Gd1—O5	69.6 (2)
C2—N2—H2A	109.3	O11—Gd1—O5	80.69 (8)
Cu2—N2—H2A	109.3	O3—Gd1—O5	72.08 (7)
C2—N2—H2B	109.3	O13B—Gd1—O5	131.3 (3)
Cu2—N2—H2B	109.3	O12B—Gd1—O5	79.6 (2)
H2A—N2—H2B	108.0	O7—Gd1—O5	72.87 (7)
C3—N3—O3	115.2 (2)	O9—Gd1—O5	138.36 (7)
C3—N3—Cu3	118.8 (2)	O1—Gd1—O5	139.11 (7)
O3—N3—Cu3	125.28 (18)	O13—Gd1—C11	28.5 (3)
C4—N4—Cu3	110.4 (2)	O12—Gd1—C11	27.9 (2)
C4—N4—H4A	109.6	O11—Gd1—C11	175.0 (3)
Cu3—N4—H4A	109.6	O3—Gd1—C11	92.2 (3)
C4—N4—H4B	109.6	O7—Gd1—C11	89.8 (3)
Cu3—N4—H4B	109.6	O9—Gd1—C11	102.9 (2)
H4A—N4—H4B	108.1	O1—Gd1—C11	102.3 (2)
C5—N5—O5	115.2 (2)	O5—Gd1—C11	97.4 (2)
C5—N5—Cu4	117.2 (2)	O11—Gd1—C11B	171.5 (2)
O5—N5—Cu4	126.74 (18)	O3—Gd1—C11B	95.6 (2)
C6—N6—Cu4	109.20 (18)	O13B—Gd1—C11B	27.0 (2)
C6—N6—H6A	109.8	O12B—Gd1—C11B	27.1 (2)
Cu4—N6—H6A	109.8	O7—Gd1—C11B	91.4 (2)
C6—N6—H6B	109.8	O9—Gd1—C11B	95.6 (2)
Cu4—N6—H6B	109.8	O1—Gd1—C11B	96.0 (2)
H6A—N6—H6B	108.3	O5—Gd1—C11B	105.8 (2)
C7—N7—O7	115.2 (2)	N1—O1—Cu1	107.85 (16)
C7—N7—Cu5	119.6 (2)	N1—O1—Gd1	123.02 (16)
O7—N7—Cu5	125.20 (17)	Cu1—O1—Gd1	125.67 (9)
C8—N8—Cu5	110.78 (17)	C1—O2—Cu1	107.29 (17)
C8—N8—H8A	109.5	N3—O3—Cu2	107.71 (15)
Cu5—N8—H8A	109.5	N3—O3—Gd1	123.98 (15)
C8—N8—H8B	109.5	Cu2—O3—Gd1	128.20 (10)
Cu5—N8—H8B	109.5	C3—O4—Cu2	106.89 (18)
H8A—N8—H8B	108.1	N5—O5—Cu3	107.81 (16)
C9—N9—O9	115.5 (2)	N5—O5—Gd1	120.57 (16)
C9—N9—Cu1	118.54 (19)	Cu3—O5—Gd1	123.24 (10)
O9—N9—Cu1	125.47 (17)	C5—O6—Cu3	106.60 (18)
C10—N10—Cu1	110.37 (18)	N7—O7—Cu4	107.98 (15)
C10—N10—H10A	109.6	N7—O7—Gd1	124.61 (15)
Cu1—N10—H10A	109.6	Cu4—O7—Gd1	125.52 (9)
C10—N10—H10B	109.6	C7—O8—Cu4	106.96 (17)
Cu1—N10—H10B	109.6	N9—O9—Cu5	107.74 (15)
H10A—N10—H10B	108.1	N9—O9—Gd1	125.05 (15)
O17—N11—O15	122.5 (3)	Cu5—O9—Gd1	126.68 (9)
O17—N11—O16	120.9 (3)	C9—O10—Cu5	107.26 (17)
O15—N11—O16	116.5 (3)	Gd1—O11—H11A	120 (3)
N9—Cu1—O1	89.32 (9)	Gd1—O11—H11B	122 (3)
N9—Cu1—O2	172.09 (10)	H11A—O11—H11B	115 (4)
O1—Cu1—O2	85.28 (9)	N11—O15—Cu2	124.8 (2)
N9—Cu1—N10	83.70 (10)	Cu1—O18—H18A	101 (3)
O1—Cu1—N10	165.70 (11)	Cu1—O18—H18B	94 (3)
O2—Cu1—N10	100.27 (10)	H18A—O18—H18B	104 (4)
N9—Cu1—O18	91.70 (9)	Cu3—O19—H19A	105 (3)
O1—Cu1—O18	95.08 (9)	Cu3—O19—H19B	111 (3)
O2—Cu1—O18	94.54 (8)	H19A—O19—H19B	106 (4)
N10—Cu1—O18	97.58 (10)	Cu4—O20—H20A	109 (3)
N1—Cu2—O3	90.48 (9)	Cu4—O20—H20B	140 (3)
N1—Cu2—O4	168.71 (10)	H20A—O20—H20B	108 (4)
O3—Cu2—O4	85.63 (9)	H21A—O21—H21B	110 (5)
N1—Cu2—N2	82.87 (11)	H22A—O22—H22B	111 (6)
O3—Cu2—N2	173.32 (10)	Cu5—O24—H24A	109 (3)
O4—Cu2—N2	100.83 (10)	Cu5—O24—H24B	107 (3)
N1—Cu2—O15	104.38 (10)	H24A—O24—H24B	95 (4)
O3—Cu2—O15	88.22 (9)	H25A—O25—H25B	100 (4)
			
O2—C1—C2—N2	−169.6 (3)	C10—C9—N9—Cu1	−9.9 (3)
N1—C1—C2—N2	9.2 (4)	C9—C10—N10—Cu1	−1.5 (3)
O4—C3—C4—N4	178.1 (3)	C9—N9—Cu1—O1	−160.3 (2)
N3—C3—C4—N4	−2.6 (4)	O9—N9—Cu1—O1	11.1 (2)
O6—C5—C6—N6	−179.6 (3)	C9—N9—Cu1—N10	7.2 (2)
N5—C5—C6—N6	0.9 (4)	O9—N9—Cu1—N10	178.6 (2)
N7—C7—C8—N8	−3.0 (4)	C9—N9—Cu1—O18	104.6 (2)
O8—C7—C8—N8	177.5 (2)	O9—N9—Cu1—O18	−84.0 (2)
O10—C9—C10—N10	−173.5 (3)	C1—N1—O1—Cu1	−6.3 (3)
N9—C9—C10—N10	7.1 (4)	Cu2—N1—O1—Cu1	178.65 (14)
O14—C11—O12—Gd1	178.3 (11)	C1—N1—O1—Gd1	−166.34 (19)
O13—C11—O12—Gd1	−3.1 (13)	Cu2—N1—O1—Gd1	18.6 (3)
O14—C11—O13—Gd1	−178.2 (10)	N1—C1—O2—Cu1	1.7 (3)
O12—C11—O13—Gd1	3.2 (13)	C2—C1—O2—Cu1	−179.6 (2)
O14B—C11B—O12B—Gd1	−179.5 (10)	C3—N3—O3—Cu2	4.8 (3)
O13B—C11B—O12B—Gd1	−1.7 (13)	Cu3—N3—O3—Cu2	−165.22 (15)
O14B—C11B—O13B—Gd1	179.5 (10)	C3—N3—O3—Gd1	−171.5 (2)
O12B—C11B—O13B—Gd1	1.7 (13)	Cu3—N3—O3—Gd1	18.4 (3)
O2—C1—N1—O1	3.2 (4)	N3—C3—O4—Cu2	−3.9 (4)
C2—C1—N1—O1	−175.5 (2)	C4—C3—O4—Cu2	175.3 (2)
O2—C1—N1—Cu2	178.5 (2)	C5—N5—O5—Cu3	−6.9 (3)
C2—C1—N1—Cu2	−0.2 (3)	Cu4—N5—O5—Cu3	161.96 (15)
C1—C2—N2—Cu2	−13.4 (3)	C5—N5—O5—Gd1	−156.0 (2)
O4—C3—N3—O3	−0.6 (4)	Cu4—N5—O5—Gd1	12.8 (3)
C4—C3—N3—O3	−179.9 (2)	N5—C5—O6—Cu3	3.2 (4)
O4—C3—N3—Cu3	170.1 (2)	C6—C5—O6—Cu3	−176.2 (2)
C4—C3—N3—Cu3	−9.1 (4)	C7—N7—O7—Cu4	9.2 (3)
C3—C4—N4—Cu3	11.8 (3)	Cu5—N7—O7—Cu4	−171.09 (14)
O6—C5—N5—O5	2.5 (4)	C7—N7—O7—Gd1	174.27 (19)
C6—C5—N5—O5	−178.0 (2)	Cu5—N7—O7—Gd1	−6.0 (3)
O6—C5—N5—Cu4	−167.4 (2)	N7—C7—O8—Cu4	−8.2 (3)
C6—C5—N5—Cu4	12.1 (4)	C8—C7—O8—Cu4	171.2 (2)
C5—C6—N6—Cu4	−12.1 (3)	C9—N9—O9—Cu5	1.1 (3)
O8—C7—N7—O7	−0.7 (4)	Cu1—N9—O9—Cu5	−170.58 (13)
C8—C7—N7—O7	179.9 (2)	C9—N9—O9—Gd1	173.21 (19)
O8—C7—N7—Cu5	179.6 (2)	Cu1—N9—O9—Gd1	1.5 (3)
C8—C7—N7—Cu5	0.2 (4)	N9—C9—O10—Cu5	1.1 (3)
C7—C8—N8—Cu5	4.2 (3)	C10—C9—O10—Cu5	−178.2 (2)
O10—C9—N9—O9	−1.6 (4)	O17—N11—O15—Cu2	131.9 (3)
C10—C9—N9—O9	177.8 (2)	O16—N11—O15—Cu2	−50.5 (4)
O10—C9—N9—Cu1	170.7 (2)		

### Pentaaquacarbonatopentakis(glycine hydroxamato)nitratopentacopper(II)gadolinium(III) 3.5-hydrate (Complex_1) Hydrogen-bond geometry (Å, º)

**Table:**

*D*—H···*A*	*D*—H	H···*A*	*D*···*A*	*D*—H···*A*
O23—H23*A*···O16^i^	0.85 (2)	1.81 (2)	2.642 (10)	165 (10)
O23—H23*B*···O14	0.84 (2)	1.90 (2)	2.644 (10)	148 (5)
N2—H2*A*···O13^i^	0.91	2.17	3.00 (2)	151
N2—H2*A*···O13*B*^i^	0.91	2.06	2.92 (2)	157
N2—H2*B*···O16	0.91	2.25	3.068 (4)	150
N4—H4*A*···O21	0.91	2.08	2.932 (4)	155
N4—H4*B*···O23^ii^	0.91	1.91	2.604 (8)	132
N6—H6*A*···O16^iii^	0.91	2.24	3.061 (4)	150
N6—H6*B*···N5^iv^	0.91	2.53	3.268 (4)	139
N6—H6*B*···O5^iv^	0.91	2.46	3.357 (4)	171
N8—H8*A*···O7^v^	0.91	2.51	3.299 (4)	145
N8—H8*A*···O20^v^	0.91	2.39	3.005 (4)	125
N8—H8*B*···O22^vi^	0.91	2.15	3.033 (4)	163
N10—H10*A*···O17^vii^	0.91	2.22	3.025 (4)	146
N10—H10*B*···O11^viii^	0.91	2.41	3.192 (4)	145
O11—H11*A*···O25	0.84 (2)	1.84 (2)	2.662 (3)	166 (4)
O11—H11*B*···O24	0.84 (2)	1.98 (2)	2.798 (3)	167 (4)
O18—H18*A*···O4^i^	0.83 (2)	1.99 (2)	2.813 (3)	169 (4)
O18—H18*B*···O13	0.83 (2)	2.07 (2)	2.886 (14)	170 (4)
O18—H18*B*···O13*B*	0.83 (2)	1.79 (2)	2.612 (14)	169 (5)
O19—H19*A*···O8^iv^	0.84 (2)	1.89 (2)	2.719 (3)	167 (4)
O19—H19*B*···O18^ii^	0.84 (2)	2.01 (2)	2.847 (3)	176 (4)
O20—H20*A*···O10^v^	0.85 (2)	1.96 (3)	2.750 (3)	156 (5)
O20—H20*B*···O14^ix^	0.85 (2)	2.17 (2)	2.997 (9)	165 (4)
O20—H20*B*···O14*B*^ix^	0.85 (2)	2.24 (2)	3.075 (9)	169 (4)
O21—H21*A*···O6^x^	0.84 (2)	2.00 (2)	2.813 (3)	163 (5)
O21—H21*B*···O22	0.85 (2)	1.83 (2)	2.650 (5)	162 (5)
O22—H22*A*···O14^ix^	0.86 (2)	1.96 (4)	2.742 (9)	150 (6)
O22—H22*A*···O14*B*^ix^	0.86 (2)	1.89 (3)	2.730 (8)	166 (6)
O22—H22*B*···O12	0.87 (2)	1.74 (3)	2.594 (12)	168 (6)
O22—H22*B*···O14	0.87 (2)	2.49 (5)	3.137 (9)	131 (5)
O22—H22*B*···O12*B*	0.87 (2)	1.94 (2)	2.803 (11)	176 (6)
O24—H24*A*···O2^viii^	0.83 (2)	1.98 (2)	2.785 (3)	163 (4)
O24—H24*B*···O21^vi^	0.84 (2)	1.95 (2)	2.755 (3)	161 (4)
O25—H25*A*···O19	0.86 (2)	1.92 (2)	2.773 (3)	173 (5)
O25—H25*B*···O15	0.85 (2)	1.93 (2)	2.754 (4)	166 (4)

Symmetry codes: (i) −*x*+2, −*y*+1, −*z*+1; (ii) *x*−1, *y*, *z*; (iii) *x*, *y*, *z*−1; (iv) −*x*+1, −*y*, −*z*; (v) −*x*+2, −*y*, −*z*; (vi) *x*, *y*−1, *z*; (vii) *x*+1, *y*, *z*; (viii) −*x*+2, −*y*, −*z*+1; (ix) −*x*+2, −*y*+1, −*z*; (x) −*x*+1, −*y*+1, −*z*.

### Pentaaquacarbonatopentakis(glycine hydroxamato)nitratopentacopper(II)dysprosium(III) 3.28-hydrate (Complex_2) Crystal data

**Table:**

[Cu_5_Dy(C_2_H_4_N_2_O_2_)_5_(CO_3_)(NO_3_)(H_2_O)_5_]·3.28H_2_O	*Z* = 2
*M**_r_* = 1191.77	*F*(000) = 1170
Triclinic, *P*1	*D*_x_ = 2.460 Mg m^−^^3^
*a* = 11.1083 (5) Å	Mo *K*α radiation, λ = 0.71073 Å
*b* = 11.4991 (5) Å	Cell parameters from 9708 reflections
*c* = 13.2894 (6) Å	θ = 3.1–28.6°
α = 93.9235 (16)°	µ = 5.65 mm^−^^1^
β = 94.7713 (17)°	*T* = 150 K
γ = 107.1470 (17)°	Block, blue
*V* = 1608.73 (13) Å^3^	0.25 × 0.21 × 0.11 mm

### Pentaaquacarbonatopentakis(glycine hydroxamato)nitratopentacopper(II)dysprosium(III) 3.28-hydrate (Complex_2) Data collection

**Table:**

Bruker AXS D8 Quest CMOS diffractometer	8274 independent reflections
Radiation source: sealed tube X-ray source	6997 reflections with *I* > 2σ(*I*)
Triumph curved graphite crystal monochromator	*R*_int_ = 0.048
ω and phi scans	θ_max_ = 28.7°, θ_min_ = 3.1°
Absorption correction: multi-scan (SADABS; Krause *et al.*, 2015)	*h* = −14→14
*T*_min_ = 0.648, *T*_max_ = 0.754	*k* = −15→15
74286 measured reflections	*l* = −17→17

### Pentaaquacarbonatopentakis(glycine hydroxamato)nitratopentacopper(II)dysprosium(III) 3.28-hydrate (Complex_2) Refinement

**Table:**

Refinement on *F*^2^	Secondary atom site location: difference Fourier map
Least-squares matrix: full	Hydrogen site location: mixed
*R*[*F*^2^ > 2σ(*F*^2^)] = 0.031	H atoms treated by a mixture of independent and constrained refinement
*wR*(*F*^2^) = 0.076	*w* = 1/[σ^2^(*F*_o_^2^) + (0.0283*P*)^2^ + 7.9239*P*] where *P* = (*F*_o_^2^ + 2*F*_c_^2^)/3
*S* = 1.08	(Δ/σ)_max_ = 0.001
8274 reflections	Δρ_max_ = 0.99 e Å^−^^3^
564 parameters	Δρ_min_ = −1.26 e Å^−^^3^
133 restraints	Extinction correction: SHELXL-2018/3 (Sheldrick 2015), Fc^*^=kFc[1+0.001xFc^2^λ^3^/sin(2θ)]^-1/4^
Primary atom site location: structure-invariant direct methods	Extinction coefficient: 0.00073 (12)

### Pentaaquacarbonatopentakis(glycine hydroxamato)nitratopentacopper(II)dysprosium(III) 3.28-hydrate (Complex_2) Special details

**Table:**

Geometry. All esds (except the esd in the dihedral angle between two l.s. planes) are estimated using the full covariance matrix. The cell esds are taken into account individually in the estimation of esds in distances, angles and torsion angles; correlations between esds in cell parameters are only used when they are defined by crystal symmetry. An approximate (isotropic) treatment of cell esds is used for estimating esds involving l.s. planes.
Refinement. The structure is isotypic to its Gd analogue, AVP815, and was solved by isomorphous replacement. A water molecule is partially occupied, inducing disorder for the nearby carbonate anion. The two disordered moieties were restrained to have similar geometries. Uij components of ADPs for disordered atoms closer to each other than 2.0 Angstrom were restrained to be similar. The distance of the water oxygen to one of the carbonate oxygen atoms was restrained to be at least 2.75 Angstrom for the moiety that contains the water molecule. Water H atom positions were refined and O-H and H···H distances were restrained to 0.84 (2) and 1.36 (2) Angstrom, respectively. The water H atom positions of the disordered moiety were further restrained based on hydrogen bonding considerations. In the final refinement cycles the partially occupied H atoms were set to ride on their carrier oxygen atom. Subject to these conditions the occupancy ratio refined to 0.280 (14) to 0.720 (14).

### Pentaaquacarbonatopentakis(glycine hydroxamato)nitratopentacopper(II)dysprosium(III) 3.28-hydrate (Complex_2) Fractional atomic coordinates and isotropic or equivalent isotropic displacement parameters (Å^2^)

**Table:**

	*x*	*y*	*z*	*U*_iso_*/*U*_eq_	Occ. (<1)
C1	1.0262 (4)	0.3060 (4)	0.5723 (3)	0.0144 (7)	
C2	1.0110 (4)	0.3901 (4)	0.6587 (3)	0.0179 (8)	
H2C	1.008419	0.349470	0.722153	0.022*	
H2D	1.084243	0.465387	0.668060	0.022*	
C3	0.6572 (4)	0.4541 (4)	0.3859 (3)	0.0165 (8)	
C4	0.5587 (4)	0.5142 (4)	0.3520 (3)	0.0212 (9)	
H4C	0.489368	0.495117	0.396268	0.025*	
H4D	0.597517	0.604064	0.358082	0.025*	
C5	0.5288 (4)	0.2157 (4)	0.0107 (3)	0.0161 (8)	
C6	0.4890 (4)	0.1761 (4)	−0.0999 (3)	0.0191 (8)	
H6C	0.395896	0.138873	−0.110990	0.023*	
H6D	0.511706	0.248396	−0.138941	0.023*	
C7	0.8143 (4)	−0.0808 (4)	−0.0313 (3)	0.0137 (7)	
C8	0.8543 (4)	−0.1927 (4)	−0.0548 (3)	0.0167 (8)	
H8C	0.778401	−0.265253	−0.068936	0.020*	
H8D	0.899991	−0.183663	−0.116018	0.020*	
C9	1.1083 (4)	−0.0375 (4)	0.3189 (3)	0.0139 (7)	
C10	1.2074 (4)	−0.0386 (4)	0.4026 (3)	0.0174 (8)	
H10C	1.185344	−0.119834	0.429057	0.021*	
H10D	1.290439	−0.024000	0.375591	0.021*	
C11	0.999 (2)	0.391 (2)	0.165 (2)	0.022 (3)	0.280 (14)
O12	0.8801 (17)	0.350 (2)	0.1404 (16)	0.023 (3)	0.280 (14)
O13	1.045 (3)	0.334 (3)	0.2344 (18)	0.024 (3)	0.280 (14)
O14	1.0694 (15)	0.4834 (17)	0.1307 (14)	0.029 (4)	0.280 (14)
O23	1.3131 (16)	0.5344 (19)	0.198 (2)	0.111 (13)	0.280 (14)
H23A	1.298662	0.593330	0.230452	0.167*	0.280 (14)
H23B	1.243199	0.486108	0.171835	0.167*	0.280 (14)
C11B	1.0347 (8)	0.3924 (8)	0.1751 (7)	0.0201 (16)	0.720 (14)
O12B	0.9201 (8)	0.3484 (8)	0.1307 (6)	0.0274 (15)	0.720 (14)
O13B	1.0523 (12)	0.3364 (10)	0.2565 (6)	0.0217 (15)	0.720 (14)
O14B	1.1204 (8)	0.4780 (6)	0.1476 (5)	0.0304 (17)	0.720 (14)
N1	0.9464 (3)	0.2917 (3)	0.4929 (2)	0.0162 (7)	
N2	0.8921 (4)	0.4220 (4)	0.6368 (3)	0.0227 (8)	
H2A	0.907785	0.503939	0.651335	0.027*	
H2B	0.834002	0.382947	0.677305	0.027*	
N3	0.6771 (4)	0.3763 (3)	0.3186 (3)	0.0180 (7)	
N4	0.5064 (4)	0.4693 (4)	0.2446 (3)	0.0269 (9)	
H4A	0.532037	0.531124	0.204512	0.032*	
H4B	0.420256	0.445419	0.239699	0.032*	
N5	0.6108 (3)	0.1700 (3)	0.0538 (2)	0.0163 (7)	
N6	0.5516 (3)	0.0862 (3)	−0.1372 (2)	0.0163 (7)	
H6A	0.583639	0.107148	−0.196466	0.020*	
H6B	0.494036	0.010417	−0.148883	0.020*	
N7	0.8515 (3)	−0.0248 (3)	0.0586 (2)	0.0140 (6)	
N8	0.9383 (3)	−0.2107 (3)	0.0324 (2)	0.0161 (7)	
H8A	1.016697	−0.203291	0.013173	0.019*	
H8B	0.906275	−0.287305	0.051533	0.019*	
N9	1.0612 (3)	0.0525 (3)	0.3265 (2)	0.0140 (6)	
N10	1.2167 (4)	0.0571 (4)	0.4862 (3)	0.0211 (7)	
H10A	1.297672	0.107865	0.497466	0.025*	
H10B	1.196810	0.021431	0.544138	0.025*	
N11	0.5764 (4)	0.2266 (4)	0.6120 (3)	0.0249 (8)	
Cu1	1.09805 (5)	0.15432 (4)	0.45064 (3)	0.01439 (10)	
Cu2	0.82144 (5)	0.37532 (4)	0.49270 (3)	0.01460 (10)	
Cu3	0.56561 (5)	0.32746 (5)	0.19608 (4)	0.01661 (11)	
Cu4	0.69158 (5)	0.08435 (5)	−0.03239 (3)	0.01462 (10)	
Cu5	0.95212 (5)	−0.08593 (4)	0.15104 (3)	0.01321 (10)	
Dy1	0.84875 (2)	0.19510 (2)	0.24230 (2)	0.01371 (6)	
O1	0.9519 (3)	0.2074 (3)	0.4149 (2)	0.0176 (6)	
O2	1.1124 (3)	0.2513 (3)	0.5797 (2)	0.0169 (6)	
O3	0.7689 (3)	0.3219 (3)	0.3506 (2)	0.0160 (6)	
O4	0.7152 (3)	0.4811 (3)	0.4763 (2)	0.0189 (6)	
O5	0.6460 (3)	0.2048 (3)	0.1576 (2)	0.0190 (6)	
O6	0.4823 (3)	0.2928 (3)	0.0572 (2)	0.0191 (6)	
O7	0.8131 (3)	0.0785 (3)	0.0789 (2)	0.0168 (6)	
O8	0.7442 (3)	−0.0466 (3)	−0.0994 (2)	0.0158 (6)	
O9	0.9731 (3)	0.0552 (3)	0.2462 (2)	0.0145 (5)	
O10	1.0775 (3)	−0.1224 (3)	0.2439 (2)	0.0156 (6)	
O11	0.7120 (3)	0.0153 (3)	0.2935 (2)	0.0202 (6)	
H11A	0.646 (3)	0.008 (5)	0.322 (4)	0.030*	
H11B	0.720 (5)	−0.054 (3)	0.284 (4)	0.030*	
O15	0.6305 (3)	0.2204 (3)	0.5340 (3)	0.0289 (7)	
O16	0.6442 (4)	0.2556 (5)	0.6960 (3)	0.0562 (13)	
O17	0.4608 (3)	0.2031 (3)	0.6087 (3)	0.0378 (9)	
O18	1.2494 (3)	0.3207 (3)	0.3755 (2)	0.0222 (6)	
H18A	1.272 (5)	0.377 (4)	0.422 (3)	0.033*	
H18B	1.197 (4)	0.334 (5)	0.333 (3)	0.033*	
O19	0.3864 (3)	0.1929 (3)	0.2659 (2)	0.0208 (6)	
H19A	0.337 (4)	0.153 (5)	0.218 (3)	0.031*	
H19B	0.340 (5)	0.225 (5)	0.295 (4)	0.031*	
O20	0.8193 (3)	0.2509 (3)	−0.1131 (3)	0.0244 (7)	
H20A	0.848 (5)	0.225 (5)	−0.162 (3)	0.037*	
H20B	0.838 (6)	0.3269 (19)	−0.105 (5)	0.037*	
O21	0.6433 (4)	0.6160 (3)	0.0964 (3)	0.0301 (8)	
H21A	0.606 (6)	0.639 (6)	0.048 (3)	0.045*	
H21B	0.685 (5)	0.573 (5)	0.070 (5)	0.045*	
O22	0.8208 (5)	0.5167 (4)	0.0461 (3)	0.0469 (11)	
H22A	0.847 (7)	0.523 (7)	−0.012 (3)	0.070*	
H22B	0.852 (7)	0.466 (6)	0.073 (6)	0.070*	
O24	0.7778 (3)	−0.1964 (3)	0.2415 (2)	0.0181 (6)	
H24A	0.806 (5)	−0.226 (5)	0.290 (3)	0.027*	
H24B	0.739 (5)	−0.259 (3)	0.204 (3)	0.027*	
O25	0.5139 (3)	0.0557 (3)	0.3692 (3)	0.0248 (7)	
H25A	0.474 (5)	0.101 (5)	0.347 (4)	0.037*	
H25B	0.538 (6)	0.097 (5)	0.425 (3)	0.037*	

### Pentaaquacarbonatopentakis(glycine hydroxamato)nitratopentacopper(II)dysprosium(III) 3.28-hydrate (Complex_2) Atomic displacement parameters (Å^2^)

**Table:**

	*U*^11^	*U*^22^	*U*^33^	*U*^12^	*U*^13^	*U*^23^
C1	0.0170 (19)	0.0133 (18)	0.0110 (17)	0.0007 (15)	0.0037 (14)	0.0030 (14)
C2	0.024 (2)	0.0163 (19)	0.0109 (17)	0.0026 (16)	0.0017 (15)	−0.0023 (14)
C3	0.021 (2)	0.0118 (18)	0.0153 (18)	0.0030 (15)	0.0036 (15)	−0.0001 (14)
C4	0.031 (2)	0.016 (2)	0.019 (2)	0.0121 (18)	−0.0001 (17)	−0.0009 (16)
C5	0.021 (2)	0.0170 (19)	0.0109 (17)	0.0061 (16)	0.0002 (15)	0.0021 (14)
C6	0.024 (2)	0.026 (2)	0.0105 (18)	0.0138 (18)	−0.0013 (15)	−0.0009 (15)
C7	0.0146 (18)	0.0150 (18)	0.0117 (17)	0.0045 (15)	0.0026 (14)	0.0015 (14)
C8	0.022 (2)	0.0159 (19)	0.0127 (18)	0.0078 (16)	0.0009 (15)	−0.0016 (14)
C9	0.0141 (18)	0.0138 (18)	0.0132 (17)	0.0031 (14)	0.0018 (14)	0.0025 (14)
C10	0.0170 (19)	0.0191 (19)	0.0174 (19)	0.0078 (16)	0.0005 (15)	0.0011 (15)
C11	0.028 (5)	0.022 (4)	0.016 (4)	0.006 (5)	0.006 (5)	0.000 (4)
O12	0.022 (6)	0.026 (4)	0.019 (5)	0.003 (5)	0.010 (5)	0.006 (4)
O13	0.027 (5)	0.023 (4)	0.015 (5)	−0.002 (4)	0.008 (5)	0.001 (5)
O14	0.030 (7)	0.029 (6)	0.020 (6)	−0.003 (6)	0.002 (6)	0.004 (5)
O23	0.052 (13)	0.058 (13)	0.23 (4)	0.042 (11)	−0.013 (16)	−0.019 (16)
C11B	0.029 (4)	0.017 (2)	0.013 (3)	0.006 (3)	0.006 (3)	−0.002 (2)
O12B	0.031 (4)	0.026 (2)	0.022 (3)	0.001 (3)	0.007 (3)	0.0090 (19)
O13B	0.025 (3)	0.021 (2)	0.015 (4)	0.002 (2)	0.000 (3)	0.001 (3)
O14B	0.041 (4)	0.023 (3)	0.018 (3)	−0.004 (3)	0.009 (3)	−0.001 (2)
N1	0.0246 (18)	0.0153 (16)	0.0084 (15)	0.0058 (14)	0.0042 (13)	−0.0038 (12)
N2	0.027 (2)	0.026 (2)	0.0151 (17)	0.0114 (16)	−0.0011 (14)	−0.0049 (14)
N3	0.0274 (19)	0.0159 (16)	0.0140 (16)	0.0114 (14)	0.0019 (14)	0.0022 (13)
N4	0.045 (3)	0.0235 (19)	0.0176 (18)	0.0205 (18)	−0.0008 (17)	0.0010 (15)
N5	0.0189 (17)	0.0201 (17)	0.0112 (15)	0.0095 (14)	−0.0016 (13)	−0.0013 (13)
N6	0.0184 (17)	0.0213 (17)	0.0107 (15)	0.0092 (14)	0.0001 (13)	−0.0008 (13)
N7	0.0200 (17)	0.0120 (15)	0.0125 (15)	0.0088 (13)	0.0030 (13)	−0.0008 (12)
N8	0.0201 (17)	0.0165 (16)	0.0130 (15)	0.0080 (14)	0.0007 (13)	0.0002 (12)
N9	0.0139 (16)	0.0185 (16)	0.0087 (14)	0.0048 (13)	−0.0035 (12)	0.0003 (12)
N10	0.0219 (19)	0.028 (2)	0.0145 (16)	0.0113 (15)	−0.0035 (14)	0.0008 (14)
N11	0.024 (2)	0.026 (2)	0.0220 (19)	0.0033 (16)	0.0022 (15)	0.0022 (15)
Cu1	0.0158 (2)	0.0178 (2)	0.0092 (2)	0.00580 (19)	−0.00147 (17)	−0.00123 (17)
Cu2	0.0202 (2)	0.0138 (2)	0.0094 (2)	0.00534 (19)	0.00115 (18)	−0.00185 (17)
Cu3	0.0250 (3)	0.0173 (2)	0.0107 (2)	0.0125 (2)	−0.00060 (19)	−0.00092 (18)
Cu4	0.0181 (2)	0.0191 (2)	0.0083 (2)	0.00965 (19)	−0.00130 (17)	−0.00176 (17)
Cu5	0.0177 (2)	0.0126 (2)	0.0102 (2)	0.00695 (18)	−0.00060 (17)	−0.00128 (17)
Dy1	0.01860 (10)	0.01323 (9)	0.00956 (9)	0.00606 (7)	−0.00016 (6)	−0.00055 (6)
O1	0.0217 (15)	0.0213 (15)	0.0108 (13)	0.0110 (12)	−0.0017 (11)	−0.0079 (11)
O2	0.0203 (15)	0.0198 (14)	0.0106 (13)	0.0067 (12)	−0.0002 (11)	0.0003 (11)
O3	0.0210 (15)	0.0171 (14)	0.0130 (13)	0.0116 (12)	0.0002 (11)	−0.0009 (11)
O4	0.0242 (16)	0.0183 (14)	0.0143 (13)	0.0071 (12)	0.0024 (11)	−0.0016 (11)
O5	0.0279 (16)	0.0280 (16)	0.0053 (12)	0.0174 (13)	−0.0034 (11)	−0.0037 (11)
O6	0.0248 (16)	0.0220 (15)	0.0141 (13)	0.0140 (13)	−0.0017 (11)	0.0010 (11)
O7	0.0244 (15)	0.0170 (14)	0.0130 (13)	0.0145 (12)	−0.0024 (11)	−0.0041 (11)
O8	0.0198 (14)	0.0192 (14)	0.0091 (12)	0.0089 (12)	−0.0025 (10)	−0.0025 (10)
O9	0.0177 (14)	0.0164 (13)	0.0092 (12)	0.0071 (11)	−0.0055 (10)	−0.0007 (10)
O10	0.0195 (14)	0.0149 (13)	0.0136 (13)	0.0081 (11)	0.0005 (11)	−0.0012 (10)
O11	0.0186 (15)	0.0170 (14)	0.0247 (16)	0.0042 (12)	0.0051 (12)	0.0019 (12)
O15	0.0303 (18)	0.0313 (18)	0.0233 (17)	0.0047 (15)	0.0068 (14)	0.0064 (14)
O16	0.042 (2)	0.088 (4)	0.0178 (18)	−0.011 (2)	0.0019 (17)	0.003 (2)
O17	0.0249 (18)	0.0300 (19)	0.058 (3)	0.0085 (15)	0.0077 (17)	−0.0026 (17)
O18	0.0247 (17)	0.0192 (15)	0.0212 (16)	0.0075 (13)	−0.0009 (13)	−0.0087 (12)
O19	0.0212 (16)	0.0255 (16)	0.0157 (14)	0.0091 (13)	0.0000 (12)	−0.0042 (12)
O20	0.0289 (18)	0.0234 (16)	0.0233 (16)	0.0108 (14)	0.0079 (13)	0.0005 (13)
O21	0.044 (2)	0.0262 (18)	0.0204 (16)	0.0149 (16)	−0.0072 (15)	0.0016 (13)
O22	0.076 (3)	0.044 (2)	0.035 (2)	0.034 (2)	0.021 (2)	0.0123 (19)
O24	0.0230 (16)	0.0172 (14)	0.0130 (14)	0.0053 (12)	−0.0016 (11)	0.0026 (11)
O25	0.0210 (16)	0.0276 (17)	0.0270 (17)	0.0094 (13)	0.0027 (13)	0.0016 (14)

### Pentaaquacarbonatopentakis(glycine hydroxamato)nitratopentacopper(II)dysprosium(III) 3.28-hydrate (Complex_2) Geometric parameters (Å, º)

**Table:**

C1—N1	1.289 (5)	N5—Cu4	1.904 (3)
C1—O2	1.293 (5)	N6—Cu4	2.005 (3)
C1—C2	1.503 (5)	N6—H6A	0.9100
C2—N2	1.484 (6)	N6—H6B	0.9100
C2—H2C	0.9900	N7—O7	1.393 (4)
C2—H2D	0.9900	N7—Cu5	1.899 (3)
C3—O4	1.288 (5)	N8—Cu5	2.021 (3)
C3—N3	1.299 (5)	N8—H8A	0.9100
C3—C4	1.512 (6)	N8—H8B	0.9100
C4—N4	1.492 (6)	N9—O9	1.395 (4)
C4—H4C	0.9900	N9—Cu1	1.897 (3)
C4—H4D	0.9900	N10—Cu1	2.012 (4)
C5—N5	1.293 (5)	N10—H10A	0.9100
C5—O6	1.298 (5)	N10—H10B	0.9100
C5—C6	1.499 (5)	N11—O17	1.228 (5)
C6—N6	1.486 (5)	N11—O15	1.249 (5)
C6—H6C	0.9900	N11—O16	1.260 (5)
C6—H6D	0.9900	Cu1—O1	1.931 (3)
C7—N7	1.292 (5)	Cu1—O2	1.949 (3)
C7—O8	1.301 (5)	Cu1—O18	2.476 (3)
C7—C8	1.501 (5)	Cu2—O3	1.931 (3)
C8—N8	1.491 (5)	Cu2—O4	1.939 (3)
C8—H8C	0.9900	Cu2—O15	2.464 (3)
C8—H8D	0.9900	Cu3—O5	1.941 (3)
C9—N9	1.293 (5)	Cu3—O6	1.954 (3)
C9—O10	1.295 (5)	Cu3—O19	2.430 (3)
C9—C10	1.502 (5)	Cu4—O7	1.936 (3)
C10—N10	1.484 (5)	Cu4—O8	1.956 (3)
C10—H10C	0.9900	Cu4—O20	2.400 (3)
C10—H10D	0.9900	Cu5—O9	1.932 (3)
C11—O14	1.256 (15)	Cu5—O10	1.941 (3)
C11—O12	1.275 (16)	Cu5—O24	2.440 (3)
C11—O13	1.318 (16)	Dy1—O11	2.357 (3)
C11—Dy1	2.704 (19)	Dy1—O3	2.382 (3)
O12—Dy1	2.27 (2)	Dy1—O9	2.410 (3)
O13—Dy1	2.31 (3)	Dy1—O7	2.412 (3)
O23—H23A	0.8410	Dy1—O1	2.453 (3)
O23—H23B	0.8398	Dy1—O5	2.469 (3)
C11B—O14B	1.251 (8)	O11—H11A	0.83 (2)
C11B—O12B	1.295 (9)	O11—H11B	0.82 (2)
C11B—O13B	1.326 (8)	O18—H18A	0.83 (2)
C11B—Dy1	2.834 (8)	O18—H18B	0.83 (2)
O12B—Dy1	2.380 (8)	O19—H19A	0.82 (2)
O13B—Dy1	2.347 (12)	O19—H19B	0.82 (2)
N1—O1	1.387 (4)	O20—H20A	0.82 (2)
N1—Cu2	1.907 (4)	O20—H20B	0.83 (2)
N2—Cu2	1.983 (4)	O21—H21A	0.84 (2)
N2—H2A	0.9100	O21—H21B	0.84 (2)
N2—H2B	0.9100	O22—H22A	0.85 (2)
N3—O3	1.398 (4)	O22—H22B	0.85 (2)
N3—Cu3	1.905 (4)	O24—H24A	0.83 (2)
N4—Cu3	2.017 (4)	O24—H24B	0.83 (2)
N4—H4A	0.9100	O25—H25A	0.83 (2)
N4—H4B	0.9100	O25—H25B	0.83 (2)
N5—O5	1.399 (4)		
			
N1—C1—O2	123.5 (4)	O4—Cu2—O15	86.41 (12)
N1—C1—C2	115.2 (4)	N2—Cu2—O15	93.60 (15)
O2—C1—C2	121.2 (3)	N3—Cu3—O5	90.67 (13)
N2—C2—C1	109.7 (3)	N3—Cu3—O6	168.26 (15)
N2—C2—H2C	109.7	O5—Cu3—O6	85.39 (12)
C1—C2—H2C	109.7	N3—Cu3—N4	82.79 (15)
N2—C2—H2D	109.7	O5—Cu3—N4	171.97 (16)
C1—C2—H2D	109.7	O6—Cu3—N4	100.11 (14)
H2C—C2—H2D	108.2	N3—Cu3—O19	97.68 (13)
O4—C3—N3	124.3 (4)	O5—Cu3—O19	97.42 (12)
O4—C3—C4	120.1 (4)	O6—Cu3—O19	93.80 (12)
N3—C3—C4	115.6 (4)	N4—Cu3—O19	88.10 (15)
N4—C4—C3	110.0 (3)	N5—Cu4—O7	91.62 (13)
N4—C4—H4C	109.7	N5—Cu4—O8	161.94 (14)
C3—C4—H4C	109.7	O7—Cu4—O8	84.56 (11)
N4—C4—H4D	109.7	N5—Cu4—N6	83.78 (14)
C3—C4—H4D	109.7	O7—Cu4—N6	173.83 (14)
H4C—C4—H4D	108.2	O8—Cu4—N6	98.55 (13)
N5—C5—O6	123.9 (4)	N5—Cu4—O20	100.27 (13)
N5—C5—C6	116.2 (4)	O7—Cu4—O20	99.73 (12)
O6—C5—C6	119.9 (4)	O8—Cu4—O20	97.78 (12)
N6—C6—C5	111.0 (3)	N6—Cu4—O20	85.18 (13)
N6—C6—H6C	109.4	N7—Cu5—O9	89.53 (12)
C5—C6—H6C	109.4	N7—Cu5—O10	170.19 (14)
N6—C6—H6D	109.4	O9—Cu5—O10	85.47 (11)
C5—C6—H6D	109.4	N7—Cu5—N8	83.23 (14)
H6C—C6—H6D	108.0	O9—Cu5—N8	169.36 (13)
N7—C7—O8	123.7 (4)	O10—Cu5—N8	100.49 (13)
N7—C7—C8	116.3 (3)	N7—Cu5—O24	95.86 (13)
O8—C7—C8	120.0 (3)	O9—Cu5—O24	87.84 (11)
N8—C8—C7	110.4 (3)	O10—Cu5—O24	92.38 (11)
N8—C8—H8C	109.6	N8—Cu5—O24	100.61 (13)
C7—C8—H8C	109.6	O12—Dy1—O13	57.1 (6)
N8—C8—H8D	109.6	O12—Dy1—O11	148.9 (5)
C7—C8—H8D	109.6	O13—Dy1—O11	153.5 (6)
H8C—C8—H8D	108.1	O13B—Dy1—O11	148.4 (2)
N9—C9—O10	124.2 (4)	O13B—Dy1—O12B	54.7 (2)
N9—C9—C10	116.1 (3)	O11—Dy1—O12B	155.6 (2)
O10—C9—C10	119.6 (3)	O12—Dy1—O3	85.0 (5)
N10—C10—C9	110.6 (3)	O13—Dy1—O3	97.2 (8)
N10—C10—H10C	109.5	O13B—Dy1—O3	93.6 (3)
C9—C10—H10C	109.5	O11—Dy1—O3	92.12 (10)
N10—C10—H10D	109.5	O12B—Dy1—O3	93.8 (2)
C9—C10—H10D	109.5	O12—Dy1—O9	123.4 (5)
H10C—C10—H10D	108.1	O13—Dy1—O9	81.7 (8)
O14—C11—O12	123.4 (18)	O13B—Dy1—O9	80.7 (3)
O14—C11—O13	121.3 (19)	O11—Dy1—O9	75.78 (10)
O12—C11—O13	115.3 (18)	O12B—Dy1—O9	112.1 (2)
O14—C11—Dy1	179 (2)	O3—Dy1—O9	141.93 (9)
O12—C11—Dy1	56.8 (11)	O12—Dy1—O7	80.3 (5)
O13—C11—Dy1	58.5 (14)	O13—Dy1—O7	101.5 (7)
C11—O12—Dy1	95.2 (13)	O13B—Dy1—O7	107.8 (3)
C11—O13—Dy1	92.3 (16)	O11—Dy1—O7	84.36 (11)
H23A—O23—H23B	107.9	O12B—Dy1—O7	77.1 (2)
O14B—C11B—O12B	125.8 (7)	O3—Dy1—O7	144.46 (9)
O14B—C11B—O13B	122.3 (8)	O9—Dy1—O7	71.21 (9)
O12B—C11B—O13B	111.8 (7)	O12—Dy1—O1	126.0 (5)
O14B—C11B—Dy1	177.4 (6)	O13—Dy1—O1	77.8 (5)
O12B—C11B—Dy1	56.6 (4)	O13B—Dy1—O1	70.69 (19)
O13B—C11B—Dy1	55.3 (5)	O11—Dy1—O1	81.73 (11)
C11B—O12B—Dy1	96.4 (5)	O12B—Dy1—O1	122.6 (2)
C11B—O13B—Dy1	97.0 (6)	O3—Dy1—O1	71.77 (9)
C1—N1—O1	116.4 (3)	O9—Dy1—O1	70.83 (9)
C1—N1—Cu2	119.4 (3)	O7—Dy1—O1	141.70 (9)
O1—N1—Cu2	124.0 (3)	O12—Dy1—O5	68.7 (5)
C2—N2—Cu2	111.6 (3)	O13—Dy1—O5	125.6 (6)
C2—N2—H2A	109.3	O13B—Dy1—O5	130.3 (2)
Cu2—N2—H2A	109.3	O11—Dy1—O5	80.88 (11)
C2—N2—H2B	109.3	O12B—Dy1—O5	78.5 (2)
Cu2—N2—H2B	109.3	O3—Dy1—O5	71.92 (9)
H2A—N2—H2B	108.0	O9—Dy1—O5	138.36 (9)
C3—N3—O3	115.0 (3)	O7—Dy1—O5	72.60 (9)
C3—N3—Cu3	119.0 (3)	O1—Dy1—O5	138.84 (9)
O3—N3—Cu3	125.2 (2)	O12—Dy1—C11	28.0 (4)
C4—N4—Cu3	110.8 (3)	O13—Dy1—C11	29.1 (4)
C4—N4—H4A	109.5	O11—Dy1—C11	174.5 (7)
Cu3—N4—H4A	109.5	O3—Dy1—C11	91.8 (7)
C4—N4—H4B	109.5	O9—Dy1—C11	103.3 (6)
Cu3—N4—H4B	109.5	O7—Dy1—C11	90.2 (7)
H4A—N4—H4B	108.1	O1—Dy1—C11	103.2 (6)
C5—N5—O5	116.2 (3)	O5—Dy1—C11	96.7 (5)
C5—N5—Cu4	117.2 (3)	O13B—Dy1—C11B	27.67 (19)
O5—N5—Cu4	125.8 (2)	O11—Dy1—C11B	172.4 (2)
C6—N6—Cu4	109.1 (2)	O12B—Dy1—C11B	27.0 (2)
C6—N6—H6A	109.9	O3—Dy1—C11B	94.7 (2)
Cu4—N6—H6A	109.9	O9—Dy1—C11B	96.7 (2)
C6—N6—H6B	109.9	O7—Dy1—C11B	92.0 (2)
Cu4—N6—H6B	109.9	O1—Dy1—C11B	97.2 (2)
H6A—N6—H6B	108.3	O5—Dy1—C11B	104.4 (2)
C7—N7—O7	115.4 (3)	N1—O1—Cu1	107.4 (2)
C7—N7—Cu5	119.3 (3)	N1—O1—Dy1	123.3 (2)
O7—N7—Cu5	125.3 (2)	Cu1—O1—Dy1	125.28 (13)
C8—N8—Cu5	110.6 (2)	C1—O2—Cu1	107.0 (2)
C8—N8—H8A	109.5	N3—O3—Cu2	107.7 (2)
Cu5—N8—H8A	109.5	N3—O3—Dy1	124.3 (2)
C8—N8—H8B	109.5	Cu2—O3—Dy1	127.93 (13)
Cu5—N8—H8B	109.5	C3—O4—Cu2	107.0 (3)
H8A—N8—H8B	108.1	N5—O5—Cu3	107.2 (2)
C9—N9—O9	115.5 (3)	N5—O5—Dy1	121.8 (2)
C9—N9—Cu1	118.3 (3)	Cu3—O5—Dy1	123.95 (13)
O9—N9—Cu1	125.6 (2)	C5—O6—Cu3	106.9 (2)
C10—N10—Cu1	110.4 (2)	N7—O7—Cu4	108.0 (2)
C10—N10—H10A	109.6	N7—O7—Dy1	124.6 (2)
Cu1—N10—H10A	109.6	Cu4—O7—Dy1	125.53 (13)
C10—N10—H10B	109.6	C7—O8—Cu4	106.9 (2)
Cu1—N10—H10B	109.6	N9—O9—Cu5	107.8 (2)
H10A—N10—H10B	108.1	N9—O9—Dy1	124.9 (2)
O17—N11—O15	122.1 (4)	Cu5—O9—Dy1	126.77 (12)
O17—N11—O16	120.1 (4)	C9—O10—Cu5	107.0 (2)
O15—N11—O16	117.8 (4)	Dy1—O11—H11A	128 (4)
N9—Cu1—O1	89.39 (13)	Dy1—O11—H11B	125 (4)
N9—Cu1—O2	172.56 (14)	H11A—O11—H11B	107 (6)
O1—Cu1—O2	85.19 (12)	N11—O15—Cu2	125.3 (3)
N9—Cu1—N10	83.73 (14)	Cu1—O18—H18A	103 (4)
O1—Cu1—N10	165.35 (15)	Cu1—O18—H18B	97 (4)
O2—Cu1—N10	100.37 (13)	H18A—O18—H18B	111 (6)
N9—Cu1—O18	91.67 (12)	Cu3—O19—H19A	107 (4)
O1—Cu1—O18	95.28 (12)	Cu3—O19—H19B	117 (4)
O2—Cu1—O18	93.90 (11)	H19A—O19—H19B	101 (6)
N10—Cu1—O18	97.82 (14)	Cu4—O20—H20A	110 (4)
N1—Cu2—O3	90.66 (13)	Cu4—O20—H20B	136 (4)
N1—Cu2—O4	168.94 (14)	H20A—O20—H20B	114 (6)
O3—Cu2—O4	85.41 (12)	H21A—O21—H21B	106 (6)
N1—Cu2—N2	82.65 (15)	H22A—O22—H22B	106 (7)
O3—Cu2—N2	173.24 (14)	Cu5—O24—H24A	110 (4)
O4—Cu2—N2	101.00 (14)	Cu5—O24—H24B	106 (4)
N1—Cu2—O15	103.87 (14)	H24A—O24—H24B	101 (5)
O3—Cu2—O15	88.90 (12)	H25A—O25—H25B	95 (6)
			
N1—C1—C2—N2	9.0 (5)	O9—N9—Cu1—O1	11.1 (3)
O2—C1—C2—N2	−169.9 (4)	C9—N9—Cu1—N10	7.5 (3)
O4—C3—C4—N4	179.4 (4)	O9—N9—Cu1—N10	178.1 (3)
N3—C3—C4—N4	−0.7 (5)	C9—N9—Cu1—O18	105.2 (3)
N5—C5—C6—N6	1.9 (5)	O9—N9—Cu1—O18	−84.2 (3)
O6—C5—C6—N6	−178.9 (4)	C7—N7—Cu5—O9	174.1 (3)
N7—C7—C8—N8	−3.7 (5)	O7—N7—Cu5—O9	−5.5 (3)
O8—C7—C8—N8	177.5 (3)	C7—N7—Cu5—N8	1.9 (3)
N9—C9—C10—N10	8.5 (5)	O7—N7—Cu5—N8	−177.7 (3)
O10—C9—C10—N10	−173.1 (3)	C7—N7—Cu5—O24	−98.1 (3)
O14—C11—O12—Dy1	−179 (3)	O7—N7—Cu5—O24	82.2 (3)
O13—C11—O12—Dy1	−2 (3)	C1—N1—O1—Cu1	−6.8 (4)
O14—C11—O13—Dy1	179 (3)	Cu2—N1—O1—Cu1	178.22 (19)
O12—C11—O13—Dy1	2 (3)	C1—N1—O1—Dy1	−165.3 (3)
O14B—C11B—O12B—Dy1	179.0 (9)	Cu2—N1—O1—Dy1	19.7 (4)
O13B—C11B—O12B—Dy1	−1.8 (9)	N1—C1—O2—Cu1	1.8 (5)
O14B—C11B—O13B—Dy1	−178.9 (9)	C2—C1—O2—Cu1	−179.4 (3)
O12B—C11B—O13B—Dy1	1.9 (9)	C3—N3—O3—Cu2	5.3 (4)
O2—C1—N1—O1	3.6 (6)	Cu3—N3—O3—Cu2	−164.7 (2)
C2—C1—N1—O1	−175.2 (3)	C3—N3—O3—Dy1	−171.9 (3)
O2—C1—N1—Cu2	178.7 (3)	Cu3—N3—O3—Dy1	18.1 (4)
C2—C1—N1—Cu2	0.0 (5)	N3—C3—O4—Cu2	−4.7 (5)
C1—C2—N2—Cu2	−13.2 (4)	C4—C3—O4—Cu2	175.2 (3)
O4—C3—N3—O3	−0.4 (6)	C5—N5—O5—Cu3	−5.6 (4)
C4—C3—N3—O3	179.7 (3)	Cu4—N5—O5—Cu3	163.6 (2)
O4—C3—N3—Cu3	170.3 (3)	C5—N5—O5—Dy1	−157.2 (3)
C4—C3—N3—Cu3	−9.7 (5)	Cu4—N5—O5—Dy1	12.0 (4)
C3—C4—N4—Cu3	9.5 (5)	N5—C5—O6—Cu3	2.5 (5)
O6—C5—N5—O5	2.2 (6)	C6—C5—O6—Cu3	−176.5 (3)
C6—C5—N5—O5	−178.7 (3)	C7—N7—O7—Cu4	9.3 (4)
O6—C5—N5—Cu4	−168.0 (3)	Cu5—N7—O7—Cu4	−171.08 (19)
C6—C5—N5—Cu4	11.1 (5)	C7—N7—O7—Dy1	174.4 (3)
C5—C6—N6—Cu4	−12.7 (4)	Cu5—N7—O7—Dy1	−6.0 (4)
O8—C7—N7—O7	−1.1 (6)	N7—C7—O8—Cu4	−7.6 (5)
C8—C7—N7—O7	−179.8 (3)	C8—C7—O8—Cu4	171.1 (3)
O8—C7—N7—Cu5	179.3 (3)	C9—N9—O9—Cu5	1.2 (4)
C8—C7—N7—Cu5	0.5 (5)	Cu1—N9—O9—Cu5	−169.69 (19)
C7—C8—N8—Cu5	4.8 (4)	C9—N9—O9—Dy1	172.9 (3)
O10—C9—N9—O9	−0.9 (6)	Cu1—N9—O9—Dy1	2.0 (4)
C10—C9—N9—O9	177.5 (3)	N9—C9—O10—Cu5	0.1 (5)
O10—C9—N9—Cu1	170.7 (3)	C10—C9—O10—Cu5	−178.2 (3)
C10—C9—N9—Cu1	−11.0 (5)	O17—N11—O15—Cu2	134.4 (4)
C9—C10—N10—Cu1	−2.5 (4)	O16—N11—O15—Cu2	−47.2 (6)
C9—N9—Cu1—O1	−159.6 (3)		

### Pentaaquacarbonatopentakis(glycine hydroxamato)nitratopentacopper(II)dysprosium(III) 3.28-hydrate (Complex_2) Hydrogen-bond geometry (Å, º)

**Table:**

*D*—H···*A*	*D*—H	H···*A*	*D*···*A*	*D*—H···*A*
O23—H23*A*···O16^i^	0.84	1.84	2.61 (2)	150
O23—H23*B*···O14	0.84	1.95	2.657 (17)	141
N2—H2*A*···O13^i^	0.91	2.23	3.05 (3)	150
N2—H2*A*···O13*B*^i^	0.91	2.05	2.900 (11)	156
N2—H2*B*···O16	0.91	2.23	3.054 (6)	150
N4—H4*A*···O21	0.91	2.08	2.912 (6)	152
N4—H4*B*···O23^ii^	0.91	1.86	2.520 (16)	128
N6—H6*A*···O16^iii^	0.91	2.29	3.091 (6)	147
N6—H6*B*···N5^iv^	0.91	2.54	3.287 (5)	140
N6—H6*B*···O5^iv^	0.91	2.49	3.396 (5)	171
N8—H8*A*···O7^v^	0.91	2.48	3.262 (5)	144
N8—H8*A*···O20^v^	0.91	2.38	2.989 (5)	124
N8—H8*B*···O22^vi^	0.91	2.17	3.045 (6)	162
N10—H10*A*···O17^vii^	0.91	2.20	3.005 (5)	147
N10—H10*B*···O11^viii^	0.91	2.42	3.198 (5)	143
O11—H11*A*···O25	0.83 (2)	1.86 (3)	2.642 (4)	157 (6)
O11—H11*B*···O24	0.82 (2)	2.00 (3)	2.800 (4)	164 (6)
O18—H18*A*···O4^i^	0.83 (2)	2.01 (2)	2.821 (4)	165 (6)
O18—H18*B*···O13	0.83 (2)	2.05 (4)	2.87 (3)	169 (6)
O18—H18*B*···O13*B*	0.83 (2)	1.84 (3)	2.650 (10)	165 (6)
O19—H19*A*···O8^iv^	0.82 (2)	1.91 (2)	2.710 (4)	167 (6)
O19—H19*B*···O18^ii^	0.82 (2)	2.01 (2)	2.827 (4)	172 (6)
O20—H20*A*···O10^v^	0.82 (2)	1.95 (3)	2.738 (4)	160 (6)
O20—H20*B*···O14^ix^	0.83 (2)	2.18 (3)	2.973 (18)	158 (6)
O20—H20*B*···O14*B*^ix^	0.83 (2)	2.28 (3)	3.065 (7)	158 (6)
O21—H21*A*···O6^x^	0.84 (2)	1.98 (2)	2.810 (4)	174 (7)
O21—H21*B*···O22	0.84 (2)	1.85 (3)	2.660 (6)	160 (7)
O22—H22*A*···O14^ix^	0.85 (2)	1.91 (3)	2.736 (19)	166 (8)
O22—H22*A*···O14*B*^ix^	0.85 (2)	1.87 (2)	2.708 (8)	171 (8)
O22—H22*B*···O12	0.85 (2)	1.74 (4)	2.57 (2)	165 (8)
O22—H22*B*···O14	0.85 (2)	2.41 (7)	3.033 (18)	130 (7)
O22—H22*B*···O12*B*	0.85 (2)	1.90 (2)	2.753 (10)	179 (9)
O24—H24*A*···O2^viii^	0.83 (2)	1.97 (2)	2.777 (4)	165 (5)
O24—H24*B*···O21^vi^	0.83 (2)	1.95 (2)	2.767 (5)	168 (5)
O25—H25*A*···O19	0.83 (2)	1.95 (2)	2.769 (5)	168 (6)
O25—H25*B*···O15	0.83 (2)	1.94 (3)	2.752 (5)	163 (6)

Symmetry codes: (i) −*x*+2, −*y*+1, −*z*+1; (ii) *x*−1, *y*, *z*; (iii) *x*, *y*, *z*−1; (iv) −*x*+1, −*y*, −*z*; (v) −*x*+2, −*y*, −*z*; (vi) *x*, *y*−1, *z*; (vii) *x*+1, *y*, *z*; (viii) −*x*+2, −*y*, −*z*+1; (ix) −*x*+2, −*y*+1, −*z*; (x) −*x*+1, −*y*+1, −*z*.

### Pentaaquacarbonatopentakis(glycine hydroxamato)nitratopentacopper(II)holmium(III) 3.445-hydrate (Complex_3) Crystal data

**Table:**

[Cu_5_Ho(C_2_H_4_N_2_O_2_)_5_(CO_3_)(NO_3_)(H_2_O)_5_]·3.445H_2_O	*Z* = 2
*M**_r_* = 1197.21	*F*(000) = 1174.9
Triclinic, *P*1	*D*_x_ = 2.467 Mg m^−^^3^
*a* = 11.2027 (9) Å	Mo *K*α radiation, λ = 0.71073 Å
*b* = 11.4955 (9) Å	Cell parameters from 9995 reflections
*c* = 13.2467 (10) Å	θ = 2.3–33.2°
α = 94.001 (3)°	µ = 5.77 mm^−^^1^
β = 94.784 (3)°	*T* = 150 K
γ = 107.518 (3)°	Prism, blue
*V* = 1613.0 (2) Å^3^	0.44 × 0.42 × 0.28 mm

### Pentaaquacarbonatopentakis(glycine hydroxamato)nitratopentacopper(II)holmium(III) 3.445-hydrate (Complex_3) Data collection

**Table:**

Bruker AXS D8 Quest CMOS diffractometer	12306 independent reflections
Radiation source: fine focus sealed tube X-ray source	10012 reflections with *I* > 2σ(*I*)
Triumph curved graphite crystal monochromator	*R*_int_ = 0.042
Detector resolution: 10.4167 pixels mm^-1^	θ_max_ = 33.2°, θ_min_ = 2.5°
ω and phi scans	*h* = −17→17
Absorption correction: multi-scan (SADABS; Krause *et al.*, 2015)	*k* = −17→17
*T*_min_ = 0.548, *T*_max_ = 0.747	*l* = −20→20
50443 measured reflections	

### Pentaaquacarbonatopentakis(glycine hydroxamato)nitratopentacopper(II)holmium(III) 3.445-hydrate (Complex_3) Refinement

**Table:**

Refinement on *F*^2^	Primary atom site location: isomorphous structure methods
Least-squares matrix: full	Secondary atom site location: difference Fourier map
*R*[*F*^2^ > 2σ(*F*^2^)] = 0.033	Hydrogen site location: mixed
*wR*(*F*^2^) = 0.082	H atoms treated by a mixture of independent and constrained refinement
*S* = 1.05	*w* = 1/[σ^2^(*F*_o_^2^) + (0.0289*P*)^2^ + 5.881*P*] where *P* = (*F*_o_^2^ + 2*F*_c_^2^)/3
12306 reflections	(Δ/σ)_max_ = 0.001
563 parameters	Δρ_max_ = 2.80 e Å^−^^3^
139 restraints	Δρ_min_ = −2.27 e Å^−^^3^

### Pentaaquacarbonatopentakis(glycine hydroxamato)nitratopentacopper(II)holmium(III) 3.445-hydrate (Complex_3) Special details

**Table:**

Geometry. All esds (except the esd in the dihedral angle between two l.s. planes) are estimated using the full covariance matrix. The cell esds are taken into account individually in the estimation of esds in distances, angles and torsion angles; correlations between esds in cell parameters are only used when they are defined by crystal symmetry. An approximate (isotropic) treatment of cell esds is used for estimating esds involving l.s. planes.
Refinement. Solved by isomorphous replacement from its Gd analogue. A water molecule is partially occupied, inducing disorder for the nearby carbonate anion. The two disordered moieties were restrained to have similar geometries. Uij components of ADPs for disordered atoms closer to each other than 2.0 Angstrom were restrained to be similar. The distance of the water oxygen to one of the carbonate oxygen atoms was restrained to be at least 2.8 Angstrom for the moiety that contains the water molecule. Water H atom positions were refined and O-H and H···H distances were restrained to 0.84 (2) and 1.36 (2) Angstrom, respectively. The water H atom positions of the disordered moiety were further restrained based on hydrogen bonding considerations. Subject to these conditions the occupancy ratio refined to 0.445 (11) to 0.555 (11).

### Pentaaquacarbonatopentakis(glycine hydroxamato)nitratopentacopper(II)holmium(III) 3.445-hydrate (Complex_3) Fractional atomic coordinates and isotropic or equivalent isotropic displacement parameters (Å^2^)

**Table:**

	*x*	*y*	*z*	*U*_iso_*/*U*_eq_	Occ. (<1)
C1	1.0280 (3)	0.3079 (3)	0.5731 (2)	0.0118 (5)	
C2	1.0125 (3)	0.3910 (3)	0.6604 (2)	0.0141 (6)	
H2C	1.009297	0.349401	0.723592	0.017*	
H2D	1.085438	0.466647	0.670787	0.017*	
C3	0.6597 (3)	0.4528 (3)	0.3871 (2)	0.0138 (5)	
C4	0.5618 (3)	0.5118 (3)	0.3530 (3)	0.0177 (6)	
H4C	0.491688	0.490089	0.395941	0.021*	
H4D	0.599580	0.602076	0.361106	0.021*	
C5	0.5303 (3)	0.2146 (3)	0.0111 (2)	0.0123 (5)	
C6	0.4901 (3)	0.1750 (3)	−0.1003 (2)	0.0173 (6)	
H6C	0.397531	0.136935	−0.111278	0.021*	
H6D	0.512297	0.247545	−0.139250	0.021*	
C7	0.8131 (3)	−0.0808 (3)	−0.0328 (2)	0.0114 (5)	
C8	0.8533 (3)	−0.1924 (3)	−0.0563 (2)	0.0140 (5)	
H8C	0.778056	−0.265608	−0.070891	0.017*	
H8D	0.898930	−0.182788	−0.117611	0.017*	
C9	1.1067 (3)	−0.0368 (3)	0.3181 (2)	0.0098 (5)	
C10	1.2053 (3)	−0.0372 (3)	0.4021 (2)	0.0139 (5)	
H10C	1.182002	−0.117768	0.429942	0.017*	
H10D	1.287142	−0.024697	0.374459	0.017*	
C11	0.9971 (13)	0.3886 (16)	0.1665 (14)	0.022 (2)	0.445 (11)
O12	0.8827 (10)	0.3498 (14)	0.1383 (12)	0.0236 (19)	0.445 (11)
O13	1.042 (3)	0.334 (2)	0.2371 (15)	0.024 (2)	0.445 (11)
O14	1.0681 (9)	0.4809 (11)	0.1285 (9)	0.030 (2)	0.445 (11)
O23	1.2942 (11)	0.5207 (9)	0.1960 (8)	0.082 (4)	0.445 (11)
H23A	1.308337	0.590634	0.233418	0.122*	0.445 (11)
H23B	1.233023	0.460780	0.173402	0.122*	0.445 (11)
C11B	1.0297 (10)	0.3906 (11)	0.1741 (10)	0.0202 (19)	0.555 (11)
O12B	0.9181 (9)	0.3477 (11)	0.1319 (10)	0.0282 (19)	0.555 (11)
O13B	1.050 (2)	0.3358 (17)	0.2541 (11)	0.0225 (19)	0.555 (11)
O14B	1.1139 (8)	0.4780 (8)	0.1452 (6)	0.0272 (17)	0.555 (11)
N1	0.9480 (3)	0.2936 (2)	0.49315 (19)	0.0136 (5)	
N2	0.8953 (3)	0.4226 (3)	0.6386 (2)	0.0216 (6)	
H2A	0.911575	0.504772	0.653058	0.026*	
H2B	0.837833	0.383792	0.679653	0.026*	
N3	0.6791 (3)	0.3750 (2)	0.3201 (2)	0.0138 (5)	
N4	0.5131 (4)	0.4698 (3)	0.2452 (2)	0.0255 (7)	
H4A	0.542034	0.532305	0.206181	0.031*	
H4B	0.427458	0.447852	0.238332	0.031*	
N5	0.6111 (3)	0.1682 (2)	0.05439 (19)	0.0122 (5)	
N6	0.5523 (3)	0.0857 (3)	−0.13800 (19)	0.0136 (5)	
H6A	0.584500	0.107440	−0.197228	0.016*	
H6B	0.495105	0.009616	−0.150397	0.016*	
N7	0.8501 (3)	−0.0247 (2)	0.05795 (19)	0.0109 (4)	
N8	0.9364 (3)	−0.2099 (2)	0.0309 (2)	0.0141 (5)	
H8A	1.014323	−0.201893	0.011654	0.017*	
H8B	0.904566	−0.286957	0.049667	0.017*	
N9	1.0597 (2)	0.0533 (2)	0.32597 (19)	0.0110 (4)	
N10	1.2179 (3)	0.0612 (3)	0.4850 (2)	0.0185 (5)	
H10A	1.298457	0.112497	0.494020	0.022*	
H10B	1.200453	0.027405	0.544288	0.022*	
N11	0.5792 (3)	0.2265 (3)	0.6124 (2)	0.0229 (6)	
Cu1	1.09880 (4)	0.15681 (3)	0.44997 (3)	0.01166 (7)	
Cu2	0.82339 (4)	0.37481 (3)	0.49405 (3)	0.01165 (7)	
Cu3	0.56785 (4)	0.32620 (4)	0.19698 (3)	0.01405 (8)	
Cu4	0.69119 (4)	0.08365 (4)	−0.03271 (3)	0.01185 (7)	
Cu5	0.95011 (4)	−0.08588 (3)	0.15039 (3)	0.01067 (7)	
Ho1	0.84737 (2)	0.19371 (2)	0.24296 (2)	0.01295 (4)	
O1	0.9539 (2)	0.2094 (2)	0.41456 (17)	0.0171 (5)	
O2	1.1136 (2)	0.2531 (2)	0.57996 (16)	0.0139 (4)	
O3	0.7699 (2)	0.3202 (2)	0.35211 (16)	0.0136 (4)	
O4	0.7177 (2)	0.4801 (2)	0.47858 (17)	0.0150 (4)	
O5	0.6451 (2)	0.2021 (2)	0.15826 (16)	0.0159 (4)	
O6	0.4839 (2)	0.2914 (2)	0.05838 (17)	0.0159 (4)	
O7	0.8119 (2)	0.0781 (2)	0.07896 (17)	0.0143 (4)	
O8	0.7438 (2)	−0.0459 (2)	−0.10037 (16)	0.0142 (4)	
O9	0.9714 (2)	0.0553 (2)	0.24613 (16)	0.0120 (4)	
O10	1.0747 (2)	−0.1222 (2)	0.24305 (17)	0.0126 (4)	
O11	0.7125 (2)	0.0137 (2)	0.2961 (2)	0.0176 (5)	
H11A	0.647 (3)	0.013 (4)	0.320 (4)	0.026*	
H11B	0.718 (5)	−0.054 (2)	0.280 (4)	0.026*	
O15	0.6333 (3)	0.2185 (3)	0.5342 (2)	0.0257 (6)	
O16	0.6461 (4)	0.2526 (4)	0.6963 (2)	0.0562 (12)	
O17	0.4642 (3)	0.2061 (3)	0.6079 (3)	0.0366 (7)	
O18	1.2491 (3)	0.3228 (2)	0.3739 (2)	0.0212 (5)	
H18A	1.265 (5)	0.370 (4)	0.426 (2)	0.032*	
H18B	1.193 (4)	0.332 (5)	0.333 (3)	0.032*	
O19	0.3874 (2)	0.1934 (2)	0.26731 (19)	0.0187 (5)	
H19A	0.343 (4)	0.151 (4)	0.218 (3)	0.028*	
H19B	0.343 (4)	0.226 (4)	0.298 (3)	0.028*	
O20	0.8212 (3)	0.2497 (2)	−0.1135 (2)	0.0225 (5)	
H20A	0.839 (5)	0.217 (4)	−0.165 (3)	0.034*	
H20B	0.841 (5)	0.3242 (18)	−0.108 (4)	0.034*	
O21	0.6432 (3)	0.6168 (3)	0.0936 (2)	0.0311 (7)	
H21A	0.597 (5)	0.617 (5)	0.042 (3)	0.047*	
H21B	0.677 (5)	0.568 (4)	0.071 (4)	0.047*	
O22	0.8170 (5)	0.5170 (4)	0.0460 (3)	0.0501 (10)	
H22A	0.845 (7)	0.518 (7)	−0.013 (3)	0.075*	
H22B	0.843 (7)	0.460 (5)	0.070 (5)	0.075*	
O24	0.7767 (2)	−0.1977 (2)	0.24108 (19)	0.0173 (5)	
H24A	0.801 (4)	−0.226 (4)	0.290 (3)	0.026*	
H24B	0.737 (4)	−0.256 (3)	0.201 (3)	0.026*	
O25	0.5129 (3)	0.0538 (2)	0.3694 (2)	0.0218 (5)	
H25A	0.477 (4)	0.094 (4)	0.338 (4)	0.033*	
H25B	0.540 (5)	0.094 (4)	0.425 (2)	0.033*	

### Pentaaquacarbonatopentakis(glycine hydroxamato)nitratopentacopper(II)holmium(III) 3.445-hydrate (Complex_3) Atomic displacement parameters (Å^2^)

**Table:**

	*U*^11^	*U*^22^	*U*^33^	*U*^12^	*U*^13^	*U*^23^
C1	0.0147 (13)	0.0115 (12)	0.0078 (11)	0.0018 (10)	0.0012 (10)	0.0010 (9)
C2	0.0204 (15)	0.0121 (13)	0.0079 (12)	0.0028 (11)	0.0018 (11)	−0.0008 (10)
C3	0.0203 (15)	0.0086 (12)	0.0119 (13)	0.0034 (11)	0.0032 (11)	−0.0004 (10)
C4	0.0232 (16)	0.0141 (14)	0.0159 (14)	0.0078 (12)	−0.0007 (12)	−0.0035 (11)
C5	0.0152 (14)	0.0140 (13)	0.0081 (12)	0.0052 (11)	−0.0004 (10)	0.0010 (9)
C6	0.0209 (16)	0.0243 (16)	0.0093 (12)	0.0126 (13)	−0.0024 (11)	−0.0001 (11)
C7	0.0139 (13)	0.0129 (12)	0.0081 (12)	0.0053 (11)	0.0013 (10)	−0.0002 (9)
C8	0.0200 (15)	0.0124 (13)	0.0100 (12)	0.0068 (11)	0.0005 (11)	−0.0033 (10)
C9	0.0093 (12)	0.0113 (12)	0.0087 (11)	0.0028 (10)	0.0011 (9)	0.0015 (9)
C10	0.0138 (13)	0.0144 (13)	0.0146 (13)	0.0059 (11)	0.0001 (11)	0.0029 (10)
C11	0.025 (5)	0.024 (3)	0.017 (3)	0.009 (4)	0.005 (4)	−0.003 (3)
O12	0.022 (4)	0.026 (3)	0.022 (3)	0.004 (4)	0.003 (4)	0.008 (3)
O13	0.026 (4)	0.022 (3)	0.021 (5)	0.001 (3)	0.004 (4)	0.001 (4)
O14	0.027 (5)	0.029 (4)	0.030 (4)	0.000 (4)	0.005 (4)	0.005 (3)
O23	0.145 (10)	0.061 (6)	0.075 (7)	0.088 (7)	0.012 (7)	0.002 (5)
C11B	0.030 (4)	0.016 (2)	0.014 (3)	0.004 (3)	0.007 (3)	−0.003 (2)
O12B	0.032 (5)	0.021 (2)	0.026 (3)	−0.003 (4)	0.009 (4)	0.006 (2)
O13B	0.025 (4)	0.019 (3)	0.018 (4)	−0.002 (2)	0.001 (3)	0.002 (3)
O14B	0.032 (4)	0.021 (3)	0.019 (3)	−0.007 (3)	0.011 (3)	0.000 (2)
N1	0.0176 (12)	0.0149 (12)	0.0083 (11)	0.0056 (10)	0.0029 (9)	−0.0040 (9)
N2	0.0274 (16)	0.0282 (15)	0.0106 (12)	0.0139 (13)	−0.0014 (11)	−0.0083 (11)
N3	0.0199 (13)	0.0131 (11)	0.0095 (11)	0.0081 (10)	−0.0011 (9)	−0.0014 (9)
N4	0.046 (2)	0.0207 (14)	0.0164 (13)	0.0227 (15)	−0.0035 (13)	−0.0010 (11)
N5	0.0145 (12)	0.0167 (12)	0.0066 (10)	0.0080 (10)	−0.0024 (9)	−0.0014 (8)
N6	0.0163 (12)	0.0169 (12)	0.0076 (10)	0.0059 (10)	0.0004 (9)	−0.0004 (9)
N7	0.0153 (12)	0.0104 (10)	0.0088 (10)	0.0073 (9)	0.0004 (9)	−0.0012 (8)
N8	0.0170 (12)	0.0130 (11)	0.0130 (11)	0.0072 (10)	−0.0011 (9)	−0.0026 (9)
N9	0.0118 (11)	0.0115 (11)	0.0087 (10)	0.0035 (9)	−0.0031 (8)	−0.0011 (8)
N10	0.0191 (14)	0.0213 (14)	0.0147 (12)	0.0081 (11)	−0.0050 (10)	−0.0014 (10)
N11	0.0229 (15)	0.0218 (14)	0.0209 (14)	0.0027 (12)	0.0021 (12)	0.0007 (11)
Cu1	0.01256 (17)	0.01432 (17)	0.00767 (15)	0.00496 (14)	−0.00191 (12)	−0.00186 (12)
Cu2	0.01622 (18)	0.01112 (16)	0.00713 (15)	0.00445 (14)	0.00047 (13)	−0.00241 (12)
Cu3	0.0221 (2)	0.01470 (17)	0.00826 (16)	0.01148 (15)	−0.00165 (14)	−0.00181 (12)
Cu4	0.01516 (17)	0.01590 (17)	0.00609 (15)	0.00853 (14)	−0.00143 (12)	−0.00185 (12)
Cu5	0.01450 (17)	0.00999 (15)	0.00826 (15)	0.00602 (13)	−0.00087 (12)	−0.00170 (12)
Ho1	0.01760 (7)	0.01211 (6)	0.00911 (6)	0.00536 (5)	−0.00014 (5)	−0.00060 (4)
O1	0.0202 (11)	0.0247 (12)	0.0085 (9)	0.0135 (10)	−0.0027 (8)	−0.0079 (8)
O2	0.0158 (10)	0.0164 (10)	0.0085 (9)	0.0050 (9)	−0.0018 (8)	−0.0016 (8)
O3	0.0185 (11)	0.0150 (10)	0.0096 (9)	0.0102 (9)	−0.0013 (8)	−0.0016 (8)
O4	0.0214 (11)	0.0133 (10)	0.0101 (9)	0.0064 (9)	0.0003 (8)	−0.0037 (8)
O5	0.0229 (12)	0.0225 (11)	0.0050 (9)	0.0140 (10)	−0.0042 (8)	−0.0049 (8)
O6	0.0222 (12)	0.0193 (11)	0.0101 (10)	0.0135 (9)	−0.0016 (8)	−0.0018 (8)
O7	0.0211 (11)	0.0143 (10)	0.0106 (9)	0.0128 (9)	−0.0033 (8)	−0.0046 (8)
O8	0.0203 (11)	0.0162 (10)	0.0079 (9)	0.0098 (9)	−0.0016 (8)	−0.0023 (7)
O9	0.0159 (10)	0.0135 (10)	0.0070 (9)	0.0076 (8)	−0.0055 (7)	−0.0012 (7)
O10	0.0152 (10)	0.0116 (9)	0.0114 (9)	0.0056 (8)	0.0001 (8)	−0.0003 (7)
O11	0.0150 (11)	0.0137 (10)	0.0236 (12)	0.0037 (9)	0.0030 (9)	0.0003 (9)
O15	0.0262 (14)	0.0257 (13)	0.0224 (13)	0.0025 (11)	0.0059 (11)	0.0047 (10)
O16	0.039 (2)	0.086 (3)	0.0181 (15)	−0.0168 (19)	0.0004 (14)	0.0038 (17)
O17	0.0214 (14)	0.0286 (15)	0.059 (2)	0.0074 (12)	0.0086 (14)	−0.0025 (14)
O18	0.0242 (13)	0.0197 (12)	0.0194 (12)	0.0088 (10)	0.0004 (10)	−0.0062 (9)
O19	0.0176 (12)	0.0233 (12)	0.0142 (11)	0.0071 (10)	−0.0017 (9)	−0.0041 (9)
O20	0.0257 (13)	0.0200 (12)	0.0240 (13)	0.0097 (11)	0.0079 (10)	0.0001 (10)
O21	0.0443 (19)	0.0255 (14)	0.0248 (14)	0.0174 (13)	−0.0118 (13)	0.0005 (11)
O22	0.087 (3)	0.043 (2)	0.0364 (19)	0.037 (2)	0.026 (2)	0.0151 (16)
O24	0.0195 (12)	0.0172 (11)	0.0146 (11)	0.0050 (9)	−0.0019 (9)	0.0040 (8)
O25	0.0198 (12)	0.0216 (12)	0.0241 (13)	0.0072 (10)	0.0019 (10)	−0.0004 (10)

### Pentaaquacarbonatopentakis(glycine hydroxamato)nitratopentacopper(II)holmium(III) 3.445-hydrate (Complex_3) Geometric parameters (Å, º)

**Table:**

C1—N1	1.296 (4)	N5—Cu4	1.903 (3)
C1—O2	1.297 (4)	N6—Cu4	2.008 (3)
C1—C2	1.503 (4)	N6—H6A	0.9100
C2—N2	1.475 (5)	N6—H6B	0.9100
C2—H2C	0.9900	N7—O7	1.391 (3)
C2—H2D	0.9900	N7—Cu5	1.902 (3)
C3—N3	1.294 (4)	N8—Cu5	2.019 (3)
C3—O4	1.297 (4)	N8—H8A	0.9100
C3—C4	1.508 (5)	N8—H8B	0.9100
C4—N4	1.478 (4)	N9—O9	1.393 (3)
C4—H4C	0.9900	N9—Cu1	1.897 (2)
C4—H4D	0.9900	N10—Cu1	2.013 (3)
C5—N5	1.296 (4)	N10—H10A	0.9100
C5—O6	1.302 (4)	N10—H10B	0.9100
C5—C6	1.504 (4)	N11—O17	1.234 (4)
C6—N6	1.485 (4)	N11—O16	1.252 (5)
C6—H6C	0.9900	N11—O15	1.253 (4)
C6—H6D	0.9900	Cu1—O1	1.930 (2)
C7—N7	1.296 (4)	Cu1—O2	1.948 (2)
C7—O8	1.299 (4)	Cu1—O18	2.471 (3)
C7—C8	1.504 (4)	Cu2—O3	1.926 (2)
C8—N8	1.485 (4)	Cu2—O4	1.940 (2)
C8—H8C	0.9900	Cu2—O15	2.463 (3)
C8—H8D	0.9900	Cu3—O5	1.939 (2)
C9—O10	1.294 (3)	Cu3—O6	1.949 (2)
C9—N9	1.297 (4)	Cu3—O19	2.437 (3)
C9—C10	1.502 (4)	Cu4—O7	1.937 (2)
C10—N10	1.487 (4)	Cu4—O8	1.949 (2)
C10—H10C	0.9900	Cu4—O20	2.405 (3)
C10—H10D	0.9900	Cu5—O9	1.931 (2)
C11—O12	1.239 (11)	Cu5—O10	1.941 (2)
C11—O14	1.283 (12)	Cu5—O24	2.443 (3)
C11—O13	1.305 (13)	Ho1—O11	2.358 (2)
C11—Ho1	2.683 (14)	Ho1—O3	2.374 (2)
O12—Ho1	2.304 (16)	Ho1—O7	2.404 (2)
O13—Ho1	2.30 (3)	Ho1—O9	2.407 (2)
O23—H23A	0.8779	Ho1—O1	2.446 (2)
O23—H23B	0.8290	Ho1—O5	2.475 (2)
C11B—O14B	1.261 (9)	O11—H11A	0.817 (19)
C11B—O12B	1.262 (10)	O11—H11B	0.813 (19)
C11B—O13B	1.309 (10)	O18—H18A	0.821 (19)
C11B—Ho1	2.817 (10)	O18—H18B	0.834 (19)
O12B—Ho1	2.374 (12)	O19—H19A	0.820 (19)
O13B—Ho1	2.35 (2)	O19—H19B	0.826 (19)
N1—O1	1.391 (3)	O20—H20A	0.824 (19)
N1—Cu2	1.898 (3)	O20—H20B	0.814 (19)
N2—Cu2	1.987 (3)	O21—H21A	0.822 (19)
N2—H2A	0.9100	O21—H21B	0.827 (19)
N2—H2B	0.9100	O22—H22A	0.87 (2)
N3—O3	1.400 (3)	O22—H22B	0.86 (2)
N3—Cu3	1.908 (3)	O24—H24A	0.810 (19)
N4—Cu3	2.010 (3)	O24—H24B	0.820 (19)
N4—H4A	0.9100	O25—H25A	0.817 (19)
N4—H4B	0.9100	O25—H25B	0.826 (19)
N5—O5	1.392 (3)		
			
N1—C1—O2	123.5 (3)	O4—Cu2—O15	86.17 (10)
N1—C1—C2	115.0 (3)	N2—Cu2—O15	94.35 (12)
O2—C1—C2	121.5 (3)	N3—Cu3—O5	90.80 (10)
N2—C2—C1	109.7 (3)	N3—Cu3—O6	168.64 (12)
N2—C2—H2C	109.7	O5—Cu3—O6	85.53 (9)
C1—C2—H2C	109.7	N3—Cu3—N4	82.62 (12)
N2—C2—H2D	109.7	O5—Cu3—N4	171.72 (13)
C1—C2—H2D	109.7	O6—Cu3—N4	99.97 (11)
H2C—C2—H2D	108.2	N3—Cu3—O19	97.77 (10)
N3—C3—O4	124.0 (3)	O5—Cu3—O19	97.72 (10)
N3—C3—C4	115.6 (3)	O6—Cu3—O19	93.38 (10)
O4—C3—C4	120.4 (3)	N4—Cu3—O19	88.20 (13)
N4—C4—C3	110.2 (3)	N5—Cu4—O7	91.47 (10)
N4—C4—H4C	109.6	N5—Cu4—O8	162.00 (11)
C3—C4—H4C	109.6	O7—Cu4—O8	84.51 (9)
N4—C4—H4D	109.6	N5—Cu4—N6	83.87 (11)
C3—C4—H4D	109.6	O7—Cu4—N6	173.92 (11)
H4C—C4—H4D	108.1	O8—Cu4—N6	98.74 (10)
N5—C5—O6	123.9 (3)	N5—Cu4—O20	101.33 (10)
N5—C5—C6	116.3 (3)	O7—Cu4—O20	99.21 (10)
O6—C5—C6	119.8 (3)	O8—Cu4—O20	96.64 (9)
N6—C6—C5	110.8 (3)	N6—Cu4—O20	85.56 (11)
N6—C6—H6C	109.5	N7—Cu5—O9	89.41 (10)
C5—C6—H6C	109.5	N7—Cu5—O10	170.18 (10)
N6—C6—H6D	109.5	O9—Cu5—O10	85.63 (9)
C5—C6—H6D	109.5	N7—Cu5—N8	83.25 (11)
H6C—C6—H6D	108.1	O9—Cu5—N8	169.11 (11)
N7—C7—O8	123.2 (3)	O10—Cu5—N8	100.38 (10)
N7—C7—C8	116.1 (3)	N7—Cu5—O24	95.87 (10)
O8—C7—C8	120.7 (3)	O9—Cu5—O24	87.88 (9)
N8—C8—C7	110.6 (2)	O10—Cu5—O24	92.41 (9)
N8—C8—H8C	109.5	N8—Cu5—O24	100.83 (10)
C7—C8—H8C	109.5	O13—Ho1—O12	56.5 (4)
N8—C8—H8D	109.5	O13—Ho1—O11	152.9 (5)
C7—C8—H8D	109.5	O12—Ho1—O11	150.1 (3)
H8C—C8—H8D	108.1	O13B—Ho1—O11	148.4 (3)
O10—C9—N9	124.0 (3)	O13—Ho1—O3	96.3 (8)
O10—C9—C10	120.0 (3)	O12—Ho1—O3	86.1 (3)
N9—C9—C10	116.1 (3)	O13B—Ho1—O3	94.1 (6)
N10—C10—C9	110.9 (3)	O11—Ho1—O3	91.94 (8)
N10—C10—H10C	109.5	O13B—Ho1—O12B	53.9 (3)
C9—C10—H10C	109.5	O11—Ho1—O12B	156.3 (3)
N10—C10—H10D	109.5	O3—Ho1—O12B	93.6 (3)
C9—C10—H10D	109.5	O13—Ho1—O7	102.0 (6)
H10C—C10—H10D	108.1	O12—Ho1—O7	79.5 (4)
O12—C11—O14	120.4 (12)	O13B—Ho1—O7	106.6 (5)
O12—C11—O13	117.9 (14)	O11—Ho1—O7	85.22 (8)
O14—C11—O13	121.7 (13)	O3—Ho1—O7	144.68 (8)
O12—C11—Ho1	59.0 (8)	O12B—Ho1—O7	77.0 (3)
O14—C11—Ho1	179.1 (14)	O13—Ho1—O9	81.6 (7)
O13—C11—Ho1	58.9 (12)	O12—Ho1—O9	121.8 (3)
C11—O12—Ho1	93.5 (9)	O13B—Ho1—O9	80.2 (5)
C11—O13—Ho1	92.0 (13)	O11—Ho1—O9	76.01 (8)
H23A—O23—H23B	137.8	O3—Ho1—O9	141.82 (7)
O14B—C11B—O12B	124.9 (10)	O12B—Ho1—O9	112.0 (3)
O14B—C11B—O13B	122.2 (10)	O7—Ho1—O9	71.34 (7)
O12B—C11B—O13B	112.9 (10)	O13—Ho1—O1	76.1 (4)
O14B—C11B—Ho1	178.2 (8)	O12—Ho1—O1	124.9 (4)
O12B—C11B—Ho1	56.8 (6)	O13B—Ho1—O1	70.4 (3)
O13B—C11B—Ho1	56.1 (9)	O11—Ho1—O1	82.19 (9)
C11B—O12B—Ho1	96.8 (7)	O3—Ho1—O1	71.84 (7)
C11B—O13B—Ho1	96.4 (9)	O12B—Ho1—O1	121.4 (3)
C1—N1—O1	116.0 (3)	O7—Ho1—O1	141.85 (8)
C1—N1—Cu2	119.6 (2)	O9—Ho1—O1	70.69 (7)
O1—N1—Cu2	124.2 (2)	O13—Ho1—O5	125.9 (5)
C2—N2—Cu2	111.84 (19)	O12—Ho1—O5	69.8 (3)
C2—N2—H2A	109.2	O13B—Ho1—O5	130.1 (4)
Cu2—N2—H2A	109.2	O11—Ho1—O5	81.21 (9)
C2—N2—H2B	109.2	O3—Ho1—O5	72.17 (7)
Cu2—N2—H2B	109.2	O12B—Ho1—O5	78.7 (3)
H2A—N2—H2B	107.9	O7—Ho1—O5	72.61 (7)
C3—N3—O3	115.4 (3)	O9—Ho1—O5	138.51 (7)
C3—N3—Cu3	118.6 (2)	O1—Ho1—O5	139.56 (7)
O3—N3—Cu3	125.27 (18)	O13—Ho1—C11	29.1 (3)
C4—N4—Cu3	110.7 (2)	O12—Ho1—C11	27.5 (3)
C4—N4—H4A	109.5	O11—Ho1—C11	175.1 (4)
Cu3—N4—H4A	109.5	O3—Ho1—C11	91.9 (4)
C4—N4—H4B	109.5	O7—Ho1—C11	89.9 (4)
Cu3—N4—H4B	109.5	O9—Ho1—C11	102.7 (4)
H4A—N4—H4B	108.1	O1—Ho1—C11	101.9 (4)
C5—N5—O5	116.0 (2)	O5—Ho1—C11	97.1 (3)
C5—N5—Cu4	117.0 (2)	O13B—Ho1—C11B	27.5 (2)
O5—N5—Cu4	126.14 (19)	O11—Ho1—C11B	172.5 (3)
C6—N6—Cu4	109.22 (19)	O3—Ho1—C11B	94.7 (3)
C6—N6—H6A	109.8	O12B—Ho1—C11B	26.4 (3)
Cu4—N6—H6A	109.8	O7—Ho1—C11B	91.3 (3)
C6—N6—H6B	109.8	O9—Ho1—C11B	96.6 (3)
Cu4—N6—H6B	109.8	O1—Ho1—C11B	96.6 (3)
H6A—N6—H6B	108.3	O5—Ho1—C11B	104.1 (3)
C7—N7—O7	115.5 (2)	N1—O1—Cu1	107.69 (18)
C7—N7—Cu5	119.1 (2)	N1—O1—Ho1	123.14 (18)
O7—N7—Cu5	125.31 (18)	Cu1—O1—Ho1	125.76 (10)
C8—N8—Cu5	110.78 (19)	C1—O2—Cu1	107.06 (18)
C8—N8—H8A	109.5	N3—O3—Cu2	107.65 (16)
Cu5—N8—H8A	109.5	N3—O3—Ho1	124.14 (16)
C8—N8—H8B	109.5	Cu2—O3—Ho1	128.11 (11)
Cu5—N8—H8B	109.5	C3—O4—Cu2	106.88 (19)
H8A—N8—H8B	108.1	N5—O5—Cu3	107.34 (17)
C9—N9—O9	115.8 (2)	N5—O5—Ho1	121.38 (17)
C9—N9—Cu1	118.3 (2)	Cu3—O5—Ho1	123.44 (10)
O9—N9—Cu1	125.40 (19)	C5—O6—Cu3	106.62 (19)
C10—N10—Cu1	110.2 (2)	N7—O7—Cu4	107.95 (16)
C10—N10—H10A	109.6	N7—O7—Ho1	124.58 (17)
Cu1—N10—H10A	109.6	Cu4—O7—Ho1	125.63 (10)
C10—N10—H10B	109.6	C7—O8—Cu4	107.32 (18)
Cu1—N10—H10B	109.6	N9—O9—Cu5	107.61 (16)
H10A—N10—H10B	108.1	N9—O9—Ho1	125.18 (16)
O17—N11—O16	120.7 (4)	Cu5—O9—Ho1	126.63 (10)
O17—N11—O15	121.9 (3)	C9—O10—Cu5	107.02 (19)
O16—N11—O15	117.4 (3)	Ho1—O11—H11A	122 (3)
N9—Cu1—O1	89.22 (10)	Ho1—O11—H11B	122 (3)
N9—Cu1—O2	171.80 (11)	H11A—O11—H11B	114 (5)
O1—Cu1—O2	85.31 (9)	N11—O15—Cu2	124.4 (2)
N9—Cu1—N10	83.88 (11)	Cu1—O18—H18A	93 (4)
O1—Cu1—N10	165.90 (12)	Cu1—O18—H18B	93 (4)
O2—Cu1—N10	100.11 (11)	H18A—O18—H18B	114 (5)
N9—Cu1—O18	92.05 (10)	Cu3—O19—H19A	104 (3)
O1—Cu1—O18	95.36 (10)	Cu3—O19—H19B	118 (3)
O2—Cu1—O18	94.54 (9)	H19A—O19—H19B	108 (5)
N10—Cu1—O18	97.16 (11)	Cu4—O20—H20A	105 (4)
N1—Cu2—O3	90.61 (10)	Cu4—O20—H20B	137 (4)
N1—Cu2—O4	168.51 (11)	H20A—O20—H20B	116 (5)
O3—Cu2—O4	85.63 (9)	H21A—O21—H21B	99 (6)
N1—Cu2—N2	82.56 (12)	H22A—O22—H22B	100 (6)
O3—Cu2—N2	173.12 (11)	Cu5—O24—H24A	112 (3)
O4—Cu2—N2	100.93 (11)	Cu5—O24—H24B	104 (3)
N1—Cu2—O15	104.57 (11)	H24A—O24—H24B	106 (5)
O3—Cu2—O15	88.10 (10)	H25A—O25—H25B	105 (5)
			
N1—C1—C2—N2	8.4 (4)	C9—N9—Cu1—N10	7.6 (2)
O2—C1—C2—N2	−169.9 (3)	O9—N9—Cu1—N10	178.8 (2)
N3—C3—C4—N4	−2.7 (4)	C9—N9—Cu1—O18	104.6 (2)
O4—C3—C4—N4	177.8 (3)	O9—N9—Cu1—O18	−84.2 (2)
N5—C5—C6—N6	1.1 (4)	C1—N1—Cu2—O3	173.7 (2)
O6—C5—C6—N6	−178.8 (3)	O1—N1—Cu2—O3	−12.2 (2)
N7—C7—C8—N8	−3.6 (4)	C1—N1—Cu2—O4	103.0 (5)
O8—C7—C8—N8	177.4 (3)	O1—N1—Cu2—O4	−82.9 (6)
O10—C9—C10—N10	−174.3 (3)	C1—N1—Cu2—N2	−5.5 (3)
N9—C9—C10—N10	6.7 (4)	O1—N1—Cu2—N2	168.6 (3)
O14—C11—O12—Ho1	179.1 (16)	C1—N1—Cu2—O15	−98.1 (2)
O13—C11—O12—Ho1	−2.4 (19)	O1—N1—Cu2—O15	76.0 (2)
O12—C11—O13—Ho1	2.4 (19)	C1—N1—O1—Cu1	−6.7 (3)
O14—C11—O13—Ho1	−179.2 (16)	Cu2—N1—O1—Cu1	178.98 (15)
O14B—C11B—O12B—Ho1	179.6 (12)	C1—N1—O1—Ho1	−166.9 (2)
O13B—C11B—O12B—Ho1	−1.4 (14)	Cu2—N1—O1—Ho1	18.8 (3)
O14B—C11B—O13B—Ho1	−179.5 (12)	N1—C1—O2—Cu1	2.0 (4)
O12B—C11B—O13B—Ho1	1.4 (14)	C2—C1—O2—Cu1	−179.9 (2)
O2—C1—N1—O1	3.3 (4)	C3—N3—O3—Cu2	4.8 (3)
C2—C1—N1—O1	−174.9 (2)	Cu3—N3—O3—Cu2	−165.17 (16)
O2—C1—N1—Cu2	177.9 (2)	C3—N3—O3—Ho1	−171.8 (2)
C2—C1—N1—Cu2	−0.3 (4)	Cu3—N3—O3—Ho1	18.3 (3)
C1—C2—N2—Cu2	−12.2 (3)	N3—C3—O4—Cu2	−4.1 (4)
O4—C3—N3—O3	−0.4 (5)	C4—C3—O4—Cu2	175.3 (2)
C4—C3—N3—O3	−179.8 (3)	C5—N5—O5—Cu3	−6.4 (3)
O4—C3—N3—Cu3	170.2 (2)	Cu4—N5—O5—Cu3	162.64 (16)
C4—C3—N3—Cu3	−9.2 (4)	C5—N5—O5—Ho1	−156.4 (2)
C3—C4—N4—Cu3	12.1 (4)	Cu4—N5—O5—Ho1	12.6 (3)
O6—C5—N5—O5	1.8 (5)	N5—C5—O6—Cu3	3.7 (4)
C6—C5—N5—O5	−178.0 (3)	C6—C5—O6—Cu3	−176.4 (2)
O6—C5—N5—Cu4	−168.2 (2)	C7—N7—O7—Cu4	9.1 (3)
C6—C5—N5—Cu4	11.9 (4)	Cu5—N7—O7—Cu4	−171.06 (15)
C5—C6—N6—Cu4	−12.2 (3)	C7—N7—O7—Ho1	174.4 (2)
O8—C7—N7—O7	−0.8 (4)	Cu5—N7—O7—Ho1	−5.8 (3)
C8—C7—N7—O7	−179.8 (3)	N7—C7—O8—Cu4	−8.0 (4)
O8—C7—N7—Cu5	179.4 (2)	C8—C7—O8—Cu4	171.0 (2)
C8—C7—N7—Cu5	0.4 (4)	C9—N9—O9—Cu5	0.9 (3)
C7—C8—N8—Cu5	4.8 (3)	Cu1—N9—O9—Cu5	−170.52 (14)
O10—C9—N9—O9	−1.0 (4)	C9—N9—O9—Ho1	172.60 (19)
C10—C9—N9—O9	177.9 (2)	Cu1—N9—O9—Ho1	1.2 (3)
O10—C9—N9—Cu1	171.0 (2)	N9—C9—O10—Cu5	0.6 (3)
C10—C9—N9—Cu1	−10.0 (3)	C10—C9—O10—Cu5	−178.3 (2)
C9—C10—N10—Cu1	−0.8 (3)	O17—N11—O15—Cu2	132.2 (3)
C9—N9—Cu1—O1	−160.1 (2)	O16—N11—O15—Cu2	−50.0 (5)
O9—N9—Cu1—O1	11.1 (2)		

### Pentaaquacarbonatopentakis(glycine hydroxamato)nitratopentacopper(II)holmium(III) 3.445-hydrate (Complex_3) Hydrogen-bond geometry (Å, º)

**Table:**

*D*—H···*A*	*D*—H	H···*A*	*D*···*A*	*D*—H···*A*
O23—H23*A*···O16^i^	0.88	1.87	2.747 (11)	173
O23—H23*B*···O14	0.83	1.98	2.509 (13)	121
N2—H2*A*···O13^i^	0.91	2.17	3.00 (3)	150
N2—H2*A*···O13*B*^i^	0.91	2.05	2.90 (2)	155
N2—H2*B*···O16	0.91	2.26	3.078 (5)	149
N4—H4*A*···O21	0.91	2.07	2.914 (5)	154
N4—H4*B*···O23^ii^	0.91	1.98	2.726 (10)	138
N6—H6*A*···O16^iii^	0.91	2.25	3.060 (5)	149
N6—H6*B*···N5^iv^	0.91	2.52	3.267 (4)	139
N6—H6*B*···O5^iv^	0.91	2.46	3.359 (4)	171
N8—H8*A*···O7^v^	0.91	2.49	3.285 (4)	146
N8—H8*A*···O20^v^	0.91	2.41	3.017 (4)	124
N8—H8*B*···O22^vi^	0.91	2.17	3.048 (5)	163
N10—H10*A*···O17^vii^	0.91	2.23	3.024 (4)	145
N10—H10*B*···O11^viii^	0.91	2.39	3.183 (4)	146
O11—H11*A*···O25	0.82 (2)	1.86 (2)	2.660 (4)	165 (5)
O11—H11*B*···O24	0.81 (2)	2.00 (2)	2.801 (4)	166 (5)
O18—H18*A*···O4^i^	0.82 (2)	2.01 (3)	2.801 (3)	160 (5)
O18—H18*B*···O13	0.83 (2)	2.04 (3)	2.87 (2)	173 (5)
O18—H18*B*···O13*B*	0.83 (2)	1.84 (3)	2.671 (18)	171 (5)
O19—H19*A*···O8^iv^	0.82 (2)	1.90 (2)	2.713 (3)	174 (5)
O19—H19*B*···O18^ii^	0.83 (2)	2.02 (2)	2.840 (4)	174 (5)
O20—H20*A*···O10^v^	0.82 (2)	1.96 (3)	2.732 (3)	156 (5)
O20—H20*B*···O14^ix^	0.81 (2)	2.21 (3)	2.997 (12)	162 (5)
O20—H20*B*···O14*B*^ix^	0.81 (2)	2.27 (3)	3.060 (9)	163 (5)
O21—H21*A*···O6^x^	0.82 (2)	2.07 (3)	2.813 (4)	151 (6)
O21—H21*B*···O22	0.83 (2)	1.87 (3)	2.638 (5)	154 (6)
O22—H22*A*···O14^ix^	0.87 (2)	1.88 (3)	2.736 (12)	170 (7)
O22—H22*A*···O14*B*^ix^	0.87 (2)	1.84 (2)	2.709 (9)	173 (7)
O22—H22*B*···O12	0.86 (2)	1.74 (3)	2.600 (16)	170 (7)
O22—H22*B*···O14	0.86 (2)	2.51 (6)	3.088 (11)	125 (6)
O22—H22*B*···O12*B*	0.86 (2)	1.93 (3)	2.791 (14)	173 (7)
O24—H24*A*···O2^viii^	0.81 (2)	1.99 (2)	2.781 (3)	166 (5)
O24—H24*B*···O21^vi^	0.82 (2)	1.94 (2)	2.759 (4)	174 (5)
O25—H25*A*···O19	0.82 (2)	1.96 (2)	2.779 (4)	177 (5)
O25—H25*B*···O15	0.83 (2)	1.95 (2)	2.751 (4)	165 (5)

Symmetry codes: (i) −*x*+2, −*y*+1, −*z*+1; (ii) *x*−1, *y*, *z*; (iii) *x*, *y*, *z*−1; (iv) −*x*+1, −*y*, −*z*; (v) −*x*+2, −*y*, −*z*; (vi) *x*, *y*−1, *z*; (vii) *x*+1, *y*, *z*; (viii) −*x*+2, −*y*, −*z*+1; (ix) −*x*+2, −*y*+1, −*z*; (x) −*x*+1, −*y*+1, −*z*.
